# The effect of ω-3 polyunsaturated fatty acids on the liver lipidome, proteome and bile acid profile: parenteral *versus* enteral administration

**DOI:** 10.1038/s41598-019-54225-8

**Published:** 2019-12-13

**Authors:** Kamila Bechynska, Nikola Daskova, Nikola Vrzackova, Karel Harant, Marie Heczková, Katerina Podzimkova, Miriam Bratova, Helena Dankova, Zuzana Berkova, Vit Kosek, Jaroslav Zelenka, Jana Hajslova, Radislav Sedlacek, Jiri Suttnar, Alzbeta Hlavackova, Lenka Bartonova, Monika Cahova

**Affiliations:** 1 University of Chemistry and Technology, Prague, Czechia; 2 Center of Experimental Medicine, Institute for Clinical and Experimental Medicine, Prague, Czechia; 3 Proteomics Core Facility, BIOCEV, Faculty of Science Charles University, Prague, Czechia; 4 Czech Centre for Phenogenomics, Vestec, Czechia; 5 Institute of Hematology and Blood Transfusion, Prague, Czechia; 6 Department of Pathology, Institute for Clinical and Experimental Medicine, Prague, Czechia; 7 First Faculty of Medicine, Charles University, Prague, Czechia

**Keywords:** Metabolomics, Metabolomics

## Abstract

Parenteral nutrition (PN) is often associated with the deterioration of liver functions (PNALD). Omega-3 polyunsaturated fatty acids (PUFA) were reported to alleviate PNALD but the underlying mechanisms have not been fully unraveled yet. Using omics´ approach, we determined serum and liver lipidome, liver proteome, and liver bile acid profile as well as markers of inflammation and oxidative stress in rats administered either ω-6 PUFA based lipid emulsion (Intralipid) or ω-6/ω-3 PUFA blend (Intralipid/Omegaven) via the enteral or parenteral route. In general, we found that enteral administration of both lipid emulsions has less impact on the liver than the parenteral route. Compared with parenterally administered Intralipid, PN administration of ω-3 PUFA was associated with 1. increased content of eicosapentaenoic (EPA)- and docosahexaenoic (DHA) acids-containing lipid species; 2. higher abundance of CYP4A isoenzymes capable of bioactive lipid synthesis and the increased content of their potential products (oxidized EPA and DHA); 3. downregulation of enzymes involved CYP450 drug metabolism what may represent an adaptive mechanism counteracting the potential negative effects (enhanced ROS production) of PUFA metabolism; 4. normalized anti-oxidative capacity and 5. physiological BAs spectrum. All these findings may contribute to the explanation of ω-3 PUFA protective effects in the context of PN.

## Introduction

Parenteral nutrition (PN) provides life-saving nutritional support in situations where caloric supply via the enteral route is either not possible or cannot cover the necessary needs of the organism^1^. Nevertheless, PN does have serious adverse effects, one of which is the deterioration of liver function^2^. While the liver function abnormalities are usually normalized after discontinuation of PN, it may represent a serious risk factor in individuals receiving long term PN^3^.

The etiology of parenteral nutrition-associated liver disease (PNALD) is not well understood and is likely multifactorial^4^. Among the risk factors, intravenous fat emulsions constituents play an important role^5^. The first well-tolerated lipid emulsion (Intralipid) introduced to parenteral nutrition mixtures was based on soybean oil rich in ω-6 polyunsaturated fatty acids (PUFA)^6^. Besides their undeniable benefits comprising the dense source of non-protein calories, prevention of essential fatty acid (FA) deficiency and minimization of respiratory and metabolic stress^7^, ω-6 PUFA serves as precursors for the synthesis of pro-inflammatory cytokines and eicosanoids and Intralipid administration was associated with serious adverse effects including inflammation and oxidative stress^8^.

The introduction of fish oil rich in ω-3 PUFA into nutrition mixtures was associated with several beneficial effects in the prevention and reversal of PNALD in both infant and adult patients^9,10^. In spite of the ongoing research, the exact mechanisms that would explain why either lipid load or FA composition confers the optimal metabolic function and prevention of PNALD are not fully established yet. The proposed mechanisms include the absence of phytosterol occurring in high concentration in soybean oil and consequent normalization of bile acids (BAs) metabolism^11^, improved lipid clearance due to the PPARα activation^12^ and modulation of inflammation due to the immunomodulatory potential of eicosapentaenoic (EPA) and docosahexaenoic (DHA) acids-derived eicosanoids^13,14^.

Lipids have been recognized as essential cellular components and energy sources of living organisms. Nevertheless, recent data show that they are also a source of bioactive lipid species exerting pleiotropic effects^15^ and altered lipid composition is increasingly recognized as a signature of many disease states^16–18^. We hypothesized that not only the composition of the lipid emulsion itself but also the parenteral route of administration bypassing the physiological mechanisms of dietary fat transport and distribution would specifically influence the composition of the liver lipidome and/or proteome and may constitute one of the first triggers towards final pathology.

Therefore, the present study aimed to identify liver lipid, protein and BAs signature associated with the lipid emulsion composition and the route of administration and to identify potentially significant processes/markers that may contribute to the PN-associated liver injury. To fulfill this task, we determined liver lipidome, proteome, and BAs profile as well as markers of inflammation and oxidative stress in rats administered either ω-6 PUFA based lipid emulsion (Intralipid) or ω-6/ω-3 PUFA blend (Intralipid/Omegaven) via the enteral or parenteral route.

## Results

### Characteristics of the experimental groups

The animals were divided into five groups subjected to different nutrition regimes, i.e. control (Plasmalyte *i.v*., granules *per os*); ENIL (Plasmalyte i.v. Intralipid *per os*, granules *per os*); ENILOV (Plasmalyte i.v., Intralipid + Omegaven *per os*, granules *per os*); PNIL (nutrition mixture with Intralipid *i.v*.); PNILOV (nutrition mixture with Intralipid + Omegaven *i.v*.). Neither of the nutrition regimes resulted in the liver injury as evidenced by normal levels of serum aspartate transaminase (AST) or alanine transaminase (ALT). ω-3 PUFA administration, both enteral and parenteral, was associated with the reduction of serum triglyceride (Tg) concentration compared with controls or with animals provided only ω-6 PUFA. Parenteral administration of both emulsions led to the elevation of serum bilirubin. We did not observe any significant differences in the serum concentration of pro- (TNFα, IL-6) or anti-inflammatory (IL-10) cytokines among groups. Parenteral, but not enteral, administration of lipid emulsions resulted in the enhanced Tg accumulation in the liver but steatosis was significantly higher in PNIL than in the PNILOV group (Table 1). The histological evaluation confirmed the biochemistry data. We observed only a few cases of focal microvesicular steatosis in ENIL (2/6) or ENILOV (1/6) groups, and no signs of more severe injury. In the PNIL group, we detected liver pathology in four out of seven samples, particularly focal microvesicular steatosis (2/7), focal microvesicular steatosis combined with necrosis close to a portal tract with markers of inflammation (1/7) and metabolic changes with focal Mallory hyaline inclusions (1/7). In the PNILOV group, we found focal microvesicular steatosis (2/6) and metabolic changes with focal Mallory hyaline (2/7) (Supplementary Figure 1).

**Table 1:** Characteristics of the experimental groups.

		control	ENIL	ENILOV	PNIL	PNILOV
serum	ALT µkat. l^−1^	0.7 ± 0.2	0.7 ± 0.1	0.9 ± 0.3	0.4 ± 0.1	0.5 ± 0.2
	AST µkat. l^−1^	2.5 ± 0.7	1.8 ± 0.3	1.9 ± 0.4	2.3 ± 0.8	2.1 ± 0.5
	Tg mmol. l^−1^	0.7 ± 0.2	0.6 ± 0.2	0.4 ± 0.2^*^	0.6 ± 0.1	0.5 ± 0.3^*^
	total bilirubin µmol. l^−1^	1.5 ± 0.3	1.4 ± 0.6	1.6 ± 0.7	2.3 ± 0.4^*,#^	2.1 ± 0.6
	direct bilirubin µmol. l^−1^	0.6 ± 0.2	0.6 ± 0.3	0.5 ± 0.1	1.3 ± 0.2^*,#,‡^	0.7 ± 0.2^†^
	TNFα pg/ml	14.3 ± 6.3	12.5 ± 4.3	19.3 ± 8.7	24.4 ± 7.2	20.1 ± 6.3
	IL-6 pg/ml	17.1 ± 2.5	16.2 ± 4.3	10.5 ± 7.2	22.4 ± 6.5	19.3 ± 5.4
	IL-10 pg/ml	23 ± 31	97 ± 144	15 ± 37	142 ± 70	167 ± 142
liver	Tg µmol. mg prot^−1^	3.3 ± 1.3	3.0 ± 2.0	4.1 ± 2.1	47.8 ± 21^*,#^	13.1 ± 8.5^†^

Data are expressed as a mean ± s.d. ALT alanine transaminase; AST aspartate transaminase; Tg triglyceride. TNFα tumor necrosis factor. ^*^p < 0.05 vs control; ^#^p < 0.05 vs ENIL; ^†^p < 0.05 vs PNIL; ^‡^<0.05 vs ENILOV.

### Oxidative stress and inflammatory markers

Oxidative stress was estimated according to the malondialdehyde (MDA) concentration in the liver homogenate, antioxidant capacity, and expression of genes associated with oxidative stress (Table 2). We did not find any markers of oxidative stress in the liver of animals fed either ω-6 PUFA or ω-6/ω-3 PUFA enterally (ENIL and ENILOV groups). MDA content tended to be even lower in the ENIL group although it did not reach statistical significance (p = 0.062). Parenteral nutrition itself, irrespective of the type of the emulsion, did not elevate the MDA content in the liver but resulted in the increased expression of Hmox-1, Nqo1, and Gclc mRNA and NQO1 and GCLC protein. All these genes are known to be positively regulated in response to oxidative stress. Antioxidant capacity of liver extract was significantly lower in PNIL but not in the PNILOV group compared with control, ENIL or ENILOV groups. The mRNA expression of pro-inflammatory cytokines Ccr2 and IL-1β was significantly higher in PNIL but not in the PNILOV group compared with controls. IL-8 mRNA expression was increased in both PNIL and PNILOV groups compared with controls but it was significantly lower in PNILOV than in the PNIL group. The expression of key enzymes involved in prostaglandin synthesis Ptgs2 and Ptges did not differ among groups, the expression of Ptgis and Alox5 was below the detection limit (Table 2).

**Table 2:** Oxidative stress and inflammatory markers.

	control	ENIL	ENILOV	PNIL	PNILOV
MDA µmol. g^−1^	1.5 ± 0.8	0.5 ± 0.2	1.6 ± 2.0	1.7 ± 1.6	1.8 ± 1.5
antioxidative capacity AUC	125 ± 13	127 ± 20	119 ± 12	96 ± 12^*^	113 ± 23
Gclc mRNA	1.0 ± 0.2	1.2 ± 0.1^†,&^	0.9 ± 0.3^†^	1.6 ± 0.4^*^	2.3 ± 0.3^*^
Gclc protein	226 ± 42	253 ± 24^†,&^	234 ± 12^†,&^	415 ± 81^*^	465 ± 153^*^
Hmox1 mRNA	1.0 ± 0.1	0.7 ± 0.1^*,&^	0.8 ± 0.1^*,&^	2.3 ± 0.6^*^	2.1 ± 0.7^*^
Hmox1 protein	62.10^6^ ± 9	63 ± 5^&^	63 ± 7^&^	72 ± 8^&^	106 ± 24^*^
Nqo1 mRNA	1.0 ± 0.5	1.0 ± 0.7^†,&^	1.0 ± 0.4^†,&^	3.2 ± 0.9^*^	4.3 ± 1.7^*^
Nqo1 protein	128 ± 48	99 ± 54^†,&^	95 ± 55^†,&^	236 ± 91^*^	199 ± 52^*^
Ccr2 mRNA	1.0 ± 0.2	1.0 ± 0.3^†^	0.9 ± 0.2^†^	3.0 ± 1.7^*^	1.1 ± 0.2^†^
IL-1β mRNA	1.0 ± 0.2	0.7 ± 0.1^†^	0.9 ± 0.3^†^	1.8 ± 0.2^*^	0.9 ± 0.1^†^
IL-6 mRNA	1.0 ± 0.4	0.7 ± 0.3^&,†^	1.3 ± 0.4^&^	2.2 ± 1.0^*^	1.5 ± 0.5
IL-8 mRNA	1.0 ± 0.6	0.4 ± 0.4^*,†,&^	0.4 ± 0.1^*,†,&^	5.9 ± 2.8^*^	2.6 ± 0.9^*,†^
TNFα mRNA	1.0 ± 0.4	0.6 ± 0.3	0.7 ± 0.3	1.1 ± 0.5	0.9 ± 0.5
IL-4 mRNA	1.0 ± 0.3	1.0 ± 0.2	1.2 ± 0.5	1.0 ± 0.3	1.4 ± 0.4
Ptgs2 mRNA	1.0 ± 0.8	0.9 ± 0.4	1.2 ± 0.5	1.3 ± 0.6	0.9 ± 0.5
Ptges mRNA	1.0 ± 0.7	0.6 ± 0.4	0.7 ± 0.5	1.2 ± 0.7	0.9 ± 0.6
Ptgis mRNA	n.d.	n.d.	n.d.	n.d.	n.d.
Alox5 mRNA	n.d.	n.d.	n.d.	n.d.	n.d.

Data are expressed as a mean ± s.d. mRNA data are expressed as fold change over control, protein abundance is expressed as protein intensity. n.d. not detected. *p < 0.05 vs control; ^†^p < 0.05 vs PNIL; ^&^p < 0.05 vs PNILOV.

### Liver bile acids profile

Analysis of the bile acid profile in the liver revealed 15 bile acid species (Table 3). Unconjugated BAs were present in concentrations lower by several degrees of magnitude compared with their conjugated derivatives. Ten of the BAs were not significantly different among groups but gCDCA, DCA, gDCA and LCA content was significantly higher while tUDCA content was significantly lower in PNIL compared with all other groups including PNILOV. Neither of the BAs tested significantly differ in PNILOV and control groups.

**Table 3:** Bile acid profile in the liver.

	control	ENIL	ENILOV	PNIL	PNILOV
CA	0.02 ± 0.01	0.02 ± 0.01	0.01 ± 0.01	0.01 ± 0.01	0.01 ± 0.01
gCA	1.0 ± 0.8	0.8 ± 0.3	0.8 ± 1.1	1.0 ± 0.5	0.9 ± 0.8
tCA	78.2 ± 41.3	66.1 ± 27.3	78.1 ± 39.2	52.2 ± 19.8	32.9 ± 42.2
CDCA	0.2 ± 0.3	0.8 ± 1.0	0.3 ± 0.3	0.1 ± 0.04	0.2 ± 0.1
gCDCA	4.2 ± 3.1	1.6 ± 1.7	2.2 ± 2.1	8.6 ± 5.7^*,#,‡^	1.1 ± 0.7^†^
tCDCA	9.6 ± 3.9	8.1 ± 6.3	6.6 ± 2.9	6.5 ± 5.6	9.6 ± 4.1
αMCA	0.02 ± 0.02	0.03 ± 0.02	0.01 ± 0.01^&^	0.023 ± 0.02	0.04 ± 0.02
βMCA	0.01 ± 0.00	0.02 ± 0.01	0.01 ± 0.01	0.01 ± 0.00	0.01 ± 0.01
DCA	0.0003 ± 0.0003	0.0002 ± 0.0003	0.0001 ± 0.0000	0.0008 ± 0.0013^*,#,‡^	0.0001 ± 0.0000^†^
gDCA	2.6 ± 1.3	3.2 ± 2.4	1.5 ± 1.1	13.6 ± 6.5^*,#,‡^	6.8 ± 3.6^†^
tDCA	6.7 ± 3.2	11.2 ± 7.8	5.4 ± 3.1	6.0 ± 1.2	4.7 ± 2.6
UDCA	0.03 ± 0.01	0.05 ± 0.01	0.03 ± 0.01	0.07 ± 0.01	0.06 ± 0.01
gUDCA	0.9 ± 0.5	0.5 ± 0.2	0.24 ± 0.13	2.1 ± 1.8	1.0 ± 0.8
tUDCA	1.7 ± 0.8	1.6 ± 0.8	1.1 ± 0.8	0.6 ± 0.2^*,#^	1.0 ± 0.1^†^
LCA	0.10 ± 0.05	0.15 ± 0.01	0.05 ± 0.01	0.30 ± 0.28^*,#,‡^	0.07 ± 0.02^†^

The values are given in µM and expressed as a mean ± s.d. CA, cholic acid; gCA, glycocholic acid; tCA, taurocholic acid; CDCA, chenodeoxycholic acid; gCDCA, glycochenodeoxycholic acid; tCDCA, taurochenodeoxycholic acid; αMCA, alpha muricholic acid; βMCA, beta muricholic acid; DCA, deoxycholic acid; gDCA, glycodeoxycholic acid; tDCA, taurodeoxycholic acid; UDCA, ursodeoxycholic acid; gUDCA, glycoursodeoxycholic acid; tUDCA, tauroursodeoxycholic acid; LCA, lithocholic acid. *p < 0.05 vs control; ^#^p < 0.05 vs ENIL; ^‡^< 0.05 vs ENILOV; ^†^p < 0.05 vs PNIL; ^&^p < 0.05 vs PNILOV.

### Serum lipidome

**Figure 1:**
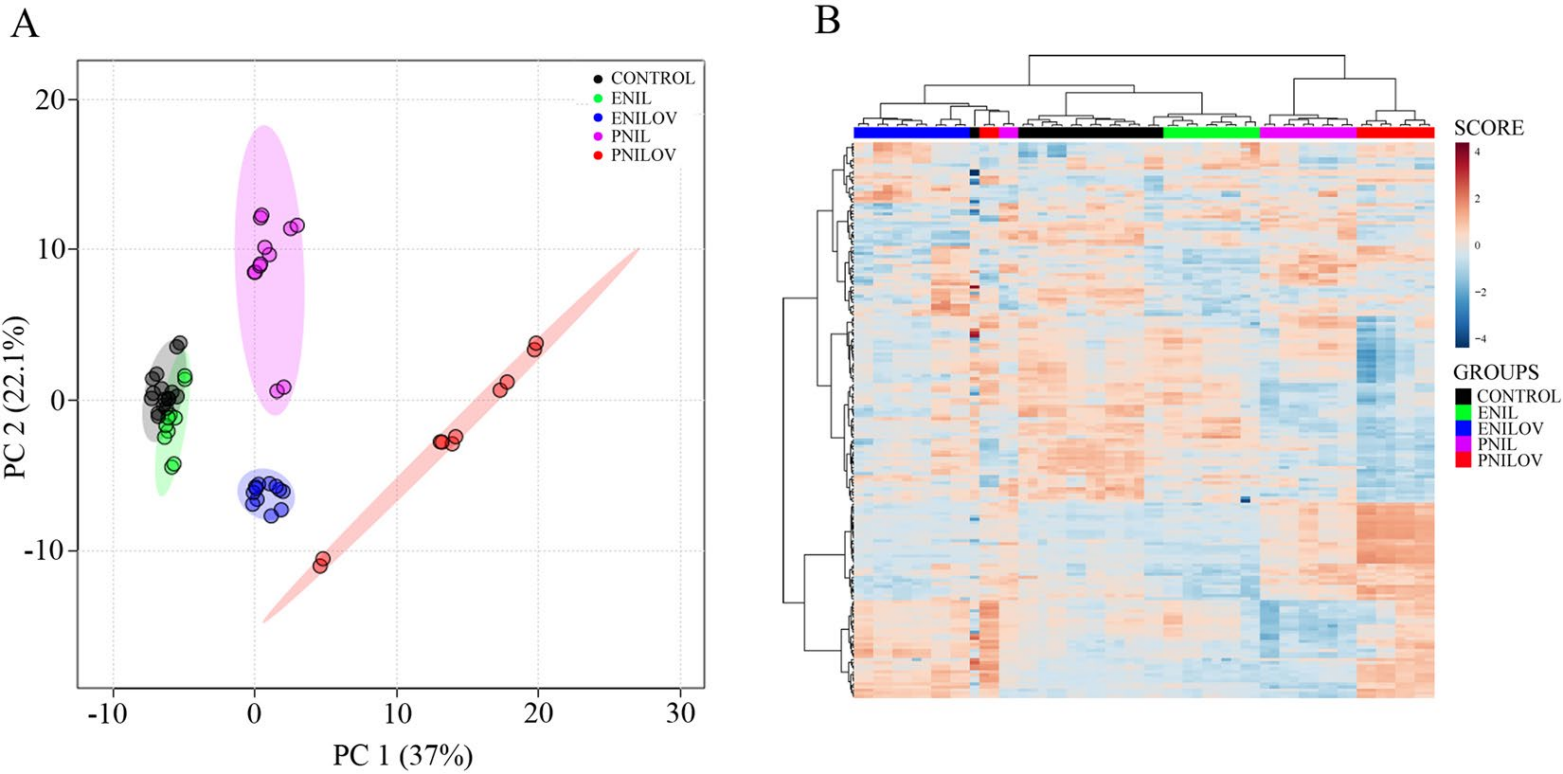
Lipidome composition in serum. (**A**) PCA score plot. Each sample was determined in a doublet. (**B**) Lipidome heatmap with the clustering dendrogram of samples. Samples are colored according to the experimental groups.

In serum, we detected 204 lipid signals confirmed by MS/MS fragmentation spectra. Using ANOVA, we filtered out 182 lipids significantly different (p < 0.01, FDR adjusted) between at least two groups. PCA analysis (Fig. 1A) identified separate clusters representing PNIL, ENILOV and PNILOV groups. Control and ENIL samples clustered into one cluster and were separated from the others. Hierarchical clustering (HC) confirmed a tendency to the separation of the groups albeit some exceptions occur (Fig. 1B).

Using supervised multivariate analysis (series of binary OPLS-DA models) based on significantly different (VIP > 1) lipids we identified eight sets of lipids most significantly contributing to the group separation. We identified partially overlapping subsets of 22, 27, 37 and 40 lipid species distinguishing ENIL, ENILOV, PNIL and PNILOV groups from controls, resp. (Supplementary Table 1). The parenteral route of lipid emulsion administration was associated with the increased content of choline- and ethanolamine-derived phospholipids containing C16 and C18 fatty acids (Supplementary Figure 2). As expected, the presence of ω-3 FA in the nutrition mixture was reflected by the increased content of EPA and DHA as well as of various lipid species containing these FAs (PNILOV > ENILOV) (Supplementary Figure 3).

### Liver lipidome

**Figure 2:**
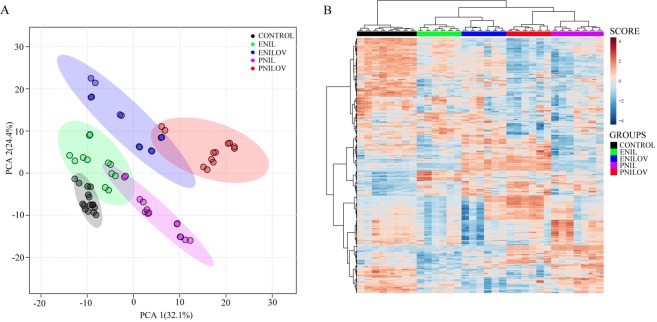
Lipidome composition in the liver. (**A**) PCA score plot. Each sample was determined in a doublet. (**B**) Lipidome heatmap with the clustering dendrogram of samples. Samples are colored according to the experimental groups.

In the liver extract, we detected 456 lipid signals confirmed by MS/MS fragmentation spectra. Using ANOVA, we filtered out 435 lipids significantly different (p < 0.01, FDR adjusted) between at least two groups. PCA scores plot (Fig. 2A) shows a separation of samples according to both the type of emulsion and the route of administration. Hierarchical clustering analysis confirmed excellent grouping of samples (Fig. 2B) with respect to these characteristics, the route of nutrition administration (enteral vs parenteral) being a superior discriminating parameter to the type of emulsion.

We further analyzed the ω-3 and ω-6 FA abundance in individual lipid classes. Regarding phospholipids, both PCA and HC analyses revealed excellent separation of the groups that suggest the combined effect of both the lipid component of the nutrition mixture and the way of administration (Supplementary Figures 4,5). Phospholipids, the main constituents of plasma membranes, were particularly enriched with EPA and DHA in PNILOV and ENILOV groups while phospholipids in the PNIL group were rich in linoleic acid, which is the prevailing component of soybean oil. Diacylglycerols and lysophospholipids containing ω-3 and ω-6 as well as respective free FA formed two separate clusters (PNILOV + ENILOV vs PNIL + ENIL + controls) but within these clusters, we did not find clear distribution pattern (Supplementary Figures 6–8). This indicates that the composition of these lipid species is dictated mainly by the dominant lipid constituent of nutrition mixture independently on the enteral or parenteral route of administration. The experimental conditions exhibited only the weak influence on the composition of the least biologically active lipid species, TAGs, (Supplementary Figure 9).

Using OPLS-DA models based on significantly different (VIP > 1) lipids we identified eight sets of lipids most significantly contributing to the group separation. As expected, the administration of lipid emulsions resulted in a significant alteration of liver lipidome. We identified partially overlapping subsets of 131, 113, 70 or 118 lipid species distinguishing ENIL, ENILOV, PNIL and PNILOV groups from controls, resp. (Supplementary Table 2).

**Figure 3:**
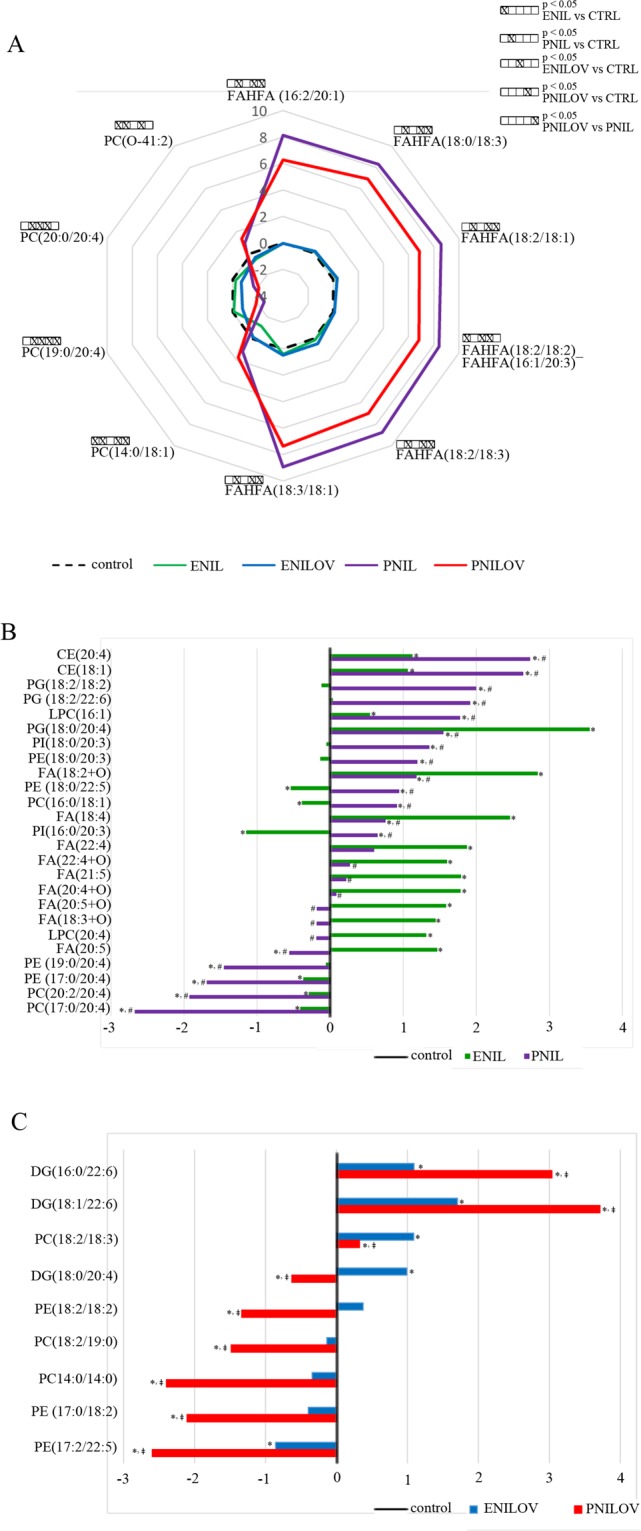
Effect of the route of administration on the relative content of selected lipid species in the liver. (**A**) all groups; (**B**) PN vs EN: Intralipid; (**C**) PN vs EN: Intralipid + Omegaven Data are expressed as log(2) fold change over median control value. *p < 0.05 vs control; ^#^p < 0.05 vs ENIL.

**Figure 4:**
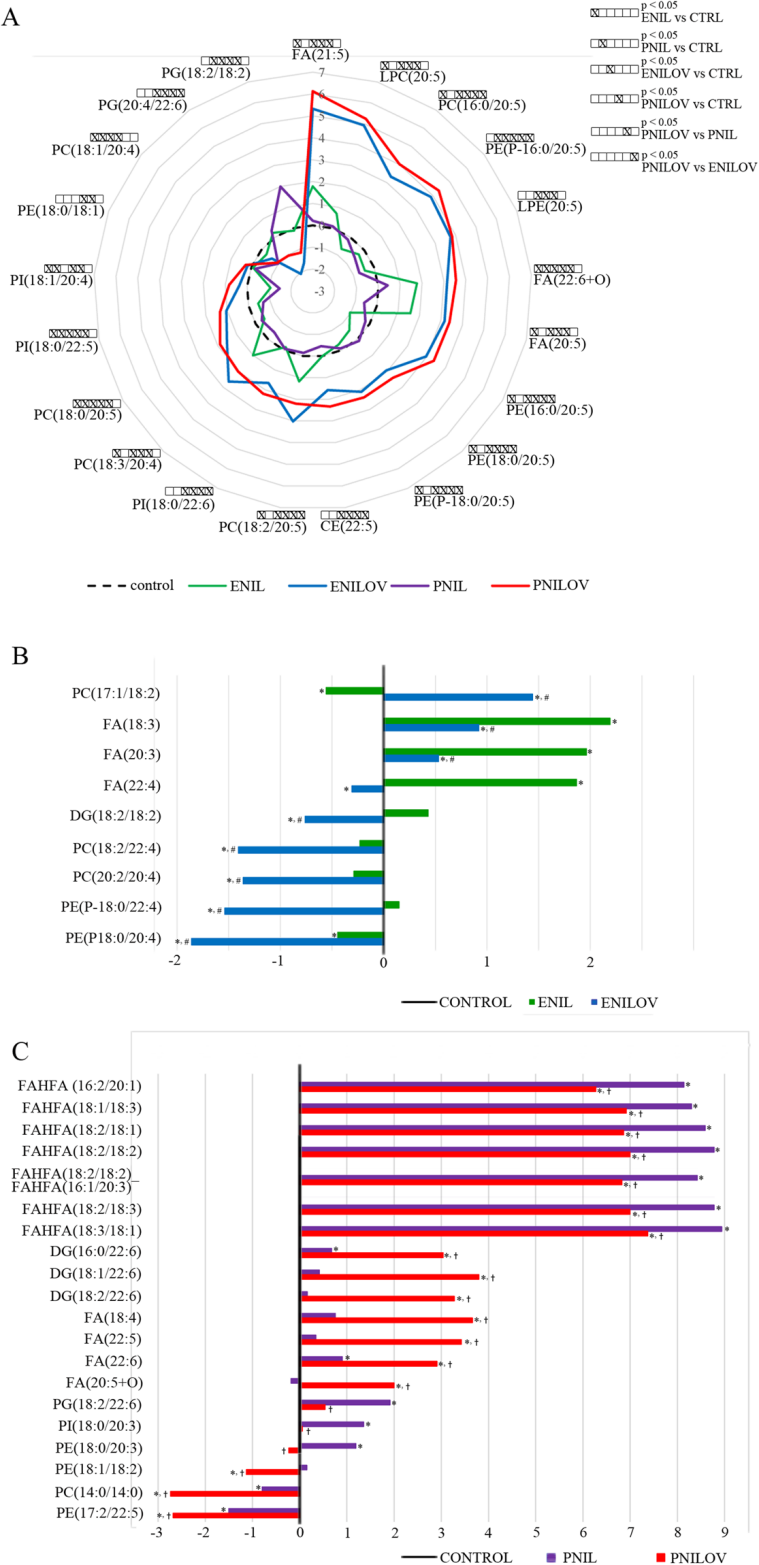
Effect of the composition of lipid emulsions on the relative content of selected lipid species in the liver. (**A**) all groups; (**B**) IL vs ILOV: enteral application; (**C**) IL vs ILOV: parenteral application. Data are expressed as log(2) fold change over median control value. *p < 0.05 vs control; ^#^p < 0.05 vs ENIL.

As the next step, we wanted to identify significant lipids characterizing the different types of emulsion or administration strategies. Figures 3 and 4 show the relative content of significant lipids (without triglycerides) expressed as log(2) fold change over mean control value. First, we evaluated the effect of the route of administration, i.e. we compared PNIL vs ENIL and PNILOV vs ENILOV groups and looked for the unique and common features. Irrespective of the type of emulsion, parenteral feeding was associated with a significantly (100- to 464-times) increased content of six fatty acid esters of hydroxy fatty acids (FAHFA), i.e. FAHFA (16:2/20:1), FAHFA (18:0/18:3), FAHFA (18:2/18:2)_FAHFA (16:1/20:3), FAHFA (18:2/18:1) and FAHFA (18:2/18:3) and FAHFA (18:3/18:1) (Fig. 3A). In both PNIL and PNILOV groups we found comparable (five times) decrease of PC(20:0/20:4). The dominant markers of ENIL compared with PNIL and control lipidome was the increased content of 18:4, 20:5 (EPA), 21:5 and 22:4 FAs, of the oxidized FAs (18:2 + O; 22:4 + O; 20:4 + O; 20:5 + O, 18:3 + O) as well as of phosphatidylglycerol (PG) containing arachidonic acid (20:4, AA). Parenteral Intralipid administration (PNIL group) was associated with significantly decreased content of phospholipids (phosphatidylethanolamines, PEs and phosphatidylcholines, PCs) containing AA mostly in combination with fatty acid with odd number of carbons and increased content of phospholipids containing linolenic acid, 22:5 FA, DHA and dihomo-γ-linoleic acid (20:3) (Fig. 3B). Parenteral feeding of ω-3 PUFA (PNILOV group) was associated with the increased concentration of diacylglycerols (DAG) containing DHA and with the decreased concentration of diacylglycerol containing AA and several other PC or PE lipid species compared with animals fed ω-3 PUFA enterally (ENILOV) or with controls (Fig. 3C).

We further examined the effect of the type of the lipid emulsion, i.e. we compared ENILOV vs ENIL and PNILOV vs PNIL groups. As expected, animals provided ω-3 PUFA exhibited the increased amount of lipid species containing EPA or DHA no matter if ω-3 PUFA was administered parenterally or *per os*. We also observed an increased amount of oxidized DHA (22:6 + O) in both ENILOV and PNILOV groups (Fig. 4A). The lipid species most discriminating between Omegaven and Intralipid emulsions administered enterally were three FAs (dihomo-γ-linoleic acid, EPA, 22:4) increased in ENIL group and PCs and plasmenyls containing AA and 22:4 FA higher in ENILOV group (Fig. 4B). In parenteral feeding, the difference between emulsions was most evident in FAHFA liver content. Albeit these compounds were dramatically increased in all parenterally fed animals, ω-3 PUFA administration resulted in their significant reduction compared with their content in the liver of animals provided only ω-6 PUFA (control ≪ PNILOV < PNIL). We also found the increased content of DHA and DAG or PG containing DHA in PNILOV compared with the PNIL group (Fig. 4C).

### Liver proteome

**Figure 5:**
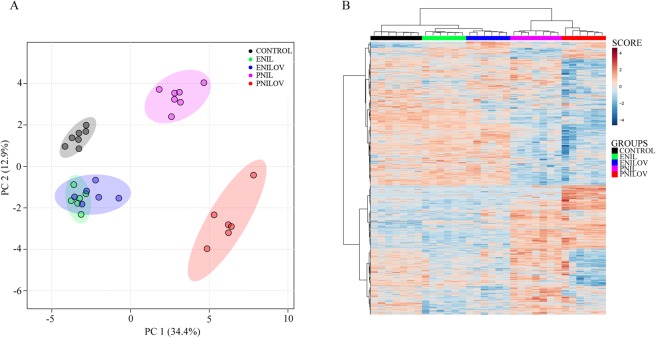
Proteome composition in the liver. (**A**) PCA score plot. (**B**) Proteome heatmap with the clustering dendrogram of samples. Samples are colored according to the experimental groups.

In the liver, we distinguished 3439 proteins. Using ANOVA, we selected 1560 proteins which abundance significantly differed (p < 0.01, FDR adjusted) between at least two groups. PCA scores plot shows clear separation of control, PNILOV and PNIL groups from each other. ENILOV and ENIL groups formed one homogenous cluster that was separated from all other groups (Fig. 5A). HCA analysis performed on all significantly different proteins (n = 1560) identified the route of nutrients administration as the dominant parameter determining the similarity/dissimilarity of the liver proteome as control, ENIL and ENILOV groups grouped into one cluster while PNIL and PNILOV groups to the other one (Fig. 5B). At the next level, the samples clustered according to either the lipid presence itself or to the type of administered lipid.

Using supervised multivariate analysis based on significantly different proteins we identified eight sets of proteins most significantly (VIP > 1) contributing to the separation of the groups (Supplementary Table 3). We further employed the DAVID database to gain insight into the potential biological function of the differentially expressed proteins obtained from the group comparisons (Supplementary Table 4). Compared with controls, enteral administration of lipid emulsions affected liver proteome less than parenteral. In the ENIL group, we found 40 differently expressed proteins; eight of them were classified into four overlapping pathways (terpenoid backbone biosynthesis; synthesis and degradation of ketone bodies; butanoate metabolism; valine, leucine and isoleucine degradation). Four of these proteins (HMGCS1, HMGCS2, ACAT1, and ACAT2) were involved in the acetoacetyl-CoA synthesis. Thirty-three proteins distinguish the ENILOV group from controls but these proteins do not cluster into any metabolic pathway. PNIL group differed from controls in 134 proteins classified into a heterogeneous spectrum of metabolic pathways including the metabolism of carbohydrates (propanoate and butanoate metabolism), amino acids (tryptophan, valine, leucine, isoleucine), fatty acids, steroid hormones, terpenoids, and retinol metabolism. Some of these pathways overlap with the ENIL group but the specific feature of the PNIL group is the deregulation (both up or down) of CYP 450 family members. In the PNILOV group, 305 proteins distinguish this group from controls. Within this subset, four metabolic pathways were identified (chemical carcinogenesis, glutathione metabolism, metabolism of xenobiotics by CYP450 and drug metabolism-CYP450). Dominating proteins in this set were isoforms of glutathione-S-transferase that were included in all these pathways. ENILOV and ENIL groups differed only in one protein (Acot1) what indicates that when administered enterally, n-3 PUFA does not significantly affect the liver proteome. Parenteral vs enteral application of ω-6 PUFA resulted in a different expression of 147 proteins. Two pathways belonging to carbohydrate metabolism (glycolysis/gluconeogenesis; pyruvate metabolism) and steroid hormone biosynthesis were identified. The groups differing in the route of administration of ω-3 PUFA (PNILOV vs ENILOV) exhibited altered expression of 198 proteins and four partially overlapping metabolic pathways (FA metabolism, FA degradation, PPARα signaling, and drug metabolism-CYP450). All these pathways were downregulated in PNILOV compared with ENILOV.

**Figure 6:**
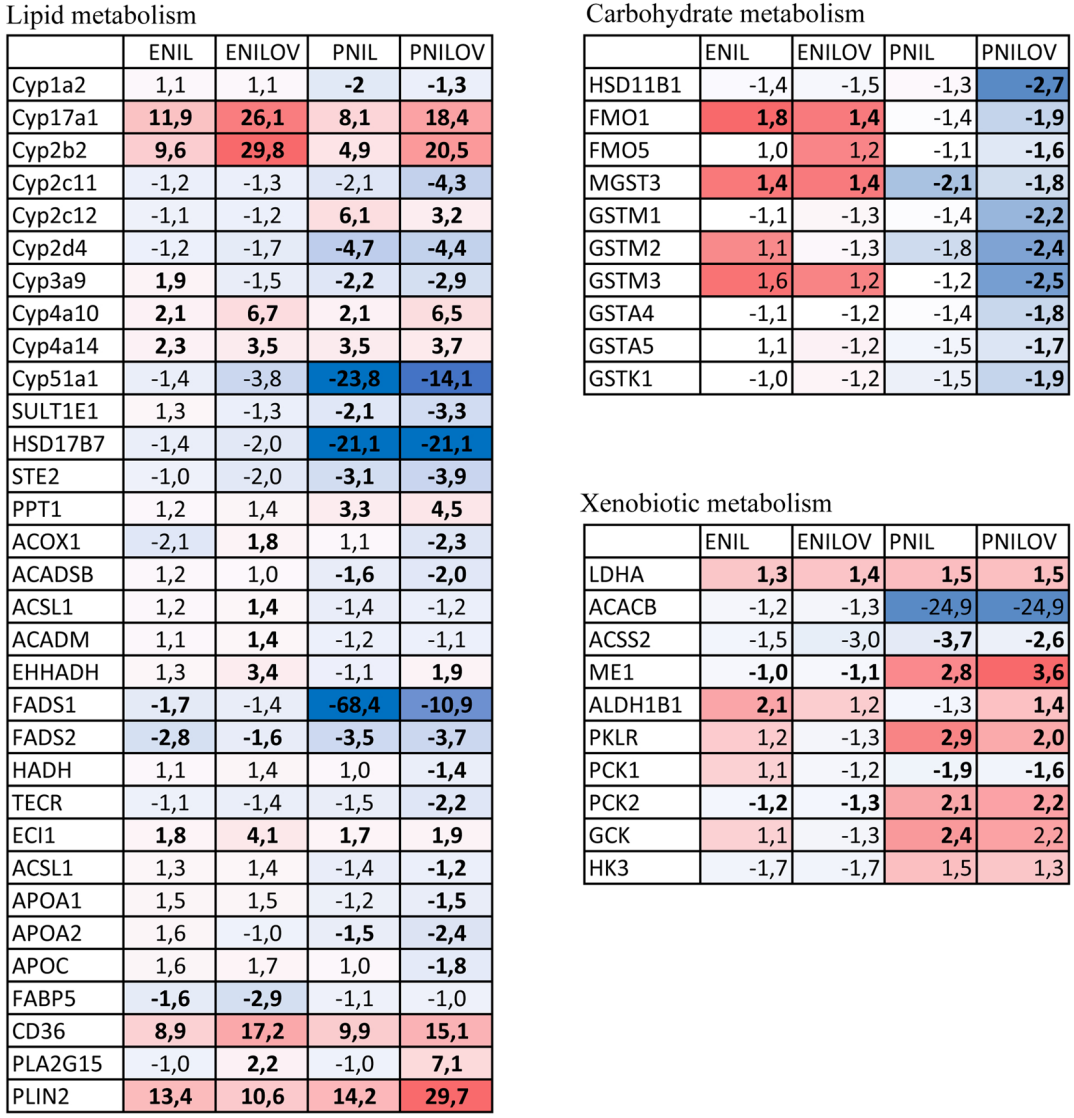
Expression of selected proteins in the liver. The values (x) represent fold change of protein intensity (experimental group to control), if x < 1 we used the equation −1/x. Statistically significant values are shown in bold. Red color: increased vs control; blue color: decreased vs control. Gene names are listed according to HGNC guidelines.

Figure 6 shows the quantitative changes in the expression of proteins included in the deregulated pathways (data are expressed as fold change over control). Most of the enzymes involved in FA degradation or FA synthesis was downregulated in PNILOV compared to other groups. Proteins involved in FA transport (CD36) and storage (PLIN2) were significantly upregulated in all experimental groups while lysosomal phospholipase A2 (PLA2G15) was upregulated only in groups provided ω-3 PUFA (PNILOV > ENILOV). Key enzymes involved in glucose metabolism (GCK, HK3, PCK2) were increased only in parenterally fed animals. All enzymes involved in the metabolism of xenobiotics or glutathione metabolism, i.e. glutathione-S-transferase isoforms, flavin-containing monooxygenases (FMO1, FMO5) and HSD11B1, were significantly downregulated in PNILOV group. Ten CYP 450 family members were deregulated due to the interventions studied in this experiment. Cyp17A1, CYP2B2, CYP4A10, and CYP4A14 were significantly upregulated in all groups (ENILOV = PNILOV ≫ ENIL > PNIL). CYP2C12 was upregulated and CYP1A2 downregulated only in parenterally fed groups but not influenced by enteral feeding. CYP2C11, CYP2D4, CYP3A9, and CYP51A1 were downregulated in all groups (control > EN feeding > PN feeding).

## Discussion

Hepatobiliary dysfunction is one of the most common complications associated with long-term dependence on parenteral nutrition. The liver injury is more severe with fat-free than with fat-containing PN, but the type of fat matters as well. Our data show that both the way of nutrient administration (enteral vs parenteral) and lipid emulsion composition (pure ω-6 vs ω-6/ω-3 mixture) significantly influence the composition of serum and liver lipidome, liver proteome and liver BAs profile in the rat model.

PUFA (AA, EPA, and DHA) are an essential source of biologically active lipids and several studies revealed that significant alterations in the content of PUFA and their metabolites occur in numerous pathophysiological conditions^19^. The first step in their synthesis is the release of FA from the *sn-2* position of membrane phospholipid or DAG followed by enzymatic conversion mediated by one of three enzymatic systems - COX, LOX or CYP450 (CYP2C, CYP2J and CYP4A families). While EPA and DHA are considered to be relatively poor substrates for COX and LOX^20^, virtually all CYP isoforms involved in AA metabolism accept a wide variety of other ω-6 and ω-3 PUFA as efficient alternative substrates^21^. Therefore, EPA and DHA compete with AA for binding and conversion by CYP 450 enzymatic system and the combination of precursor availability together with the activity of individual enzymatic systems leads to the unique combination of bioactive compounds determining the final effect.

Functionally, these compounds are extremely diverse and may exert a wide spectrum of biological properties. AA and EPA are precursors of pro-inflammatory eicosanoids (AA: 2-series prostaglandins, thromboxanes, 4-series leukotrienes; EPA: 3-series prostaglandins, 3-series thromboxanes, 5-series leukotrienes) albeit EPA gives rise to weaker pro-inflammatory agents than those derived from AA. On the other hand, all these fatty acids may be transformed into anti-inflammatory mediators like lipoxins (derived from AA), resolvins of the E-series (derived from EPA), and resolvins of the D-series, protectins, or maresins (derived from DHA)^15,19,22^. These lipokines are involved in the resolution of inflammation by several mechanisms including attenuation of the inflammatory response, induction of efferocytosis (clearance of apoptotic cells), and stimulation of macrophage migration from the site of inflammation to the peripheral lymph nodes^23^.

CYP 450-catalyzed transformation of PUFA results in the formation of hydroxy- or epoxy-PUFA^24^. These compounds serve as second messengers of various hormones and growth factors and play partially opposing roles in the regulation of vascular, renal or cardiac function and regulation of inflammation^25–30^

EPA, DHA, and AA are highly susceptible to the modification by exogenous supply. AA is derived from essential linoleic acid (LA,18:2, n-6). EPA and DHA may be synthesized from another essential FA, α-linoleic acid (ALA, 18:3, n-3) but the conversion rate is very limited^31^. Arnold *et al*. showed that oral administration of diet rich in either ω-6 or ω-3 (EPA + DHA) PUFA resulted in dramatic enrichment of liver lipidome with LA or EPA + DHA, resp^32^. Similarly, EPA/DHA supplementation caused a profound shift in the endogenous CYP 450-derived eicosanoid profile.

Our data revealed that introducing of ω-3 PUFA into lipid emulsion results in a significant enrichment of liver lipidome with EPA- and DHA-containing lipid species that may serve as substrates for bioactive lipid synthesis. The content of oxidized DHA (22:6 + O) was increased in both PNILOV and ENILOV groups while significantly elevated content of oxidized EPA (20:5 + O) was found only in PNILOV compared with other groups. Even though our methodology does not allow us to differentiate what form of oxygen modification is present (hydroperoxydation/dihydroxylation or epoxidation/hydroxylation), it still describes the occurrence of oxidized forms of free fatty acids.

In PNILOV and ENILOV groups, we further confirmed the significant upregulation of two isoenzymes from the CYP4A family that catalyze microsomal ω-oxidation and are capable of synthesis of hydroxylated derivatives of EPA or DHA^33,34^. Therefore, our data suggest that introducing ω-3 PUFA into the nutrition mixture may significantly affect both substrate availability and the CYP 450 isoenzymes activities thus altering the final CYP 450-derived eicosanoid profile towards less pro-inflammatory phenotype. This conclusion is supported by the expression pattern of pro-inflammatory cytokines (IL-8, IL-1β, Ccr2) where we found an anti-inflammatory effect of parenterally administered ω-3 PUFA. Nevertheless, it is necessary to mention that our experimental setting does not allow inferring any extensive conclusions regarding the anti-inflammatory effect of ω-3 PUFA. The general pro-inflammatory status of all animals was low what is evidenced by the normal levels of serum TNFα and IL-6 or comparable mRNA expression of TNFα and enzymes involved in prostaglandin synthesis in the liver among groups.

A characteristic feature, discriminating all PN patients from controls or enterally fed experimental groups, is an elevated content of FAHFAs in the liver. Only recently, this novel class of endogenous lipokines, branched fatty acid esters of hydroxy fatty acids, was identified^35^. These FAs are synthesized in nearly all mammalian tissues being most abundant in adipose tissue and liver, and they are also present in the blood. The biological function of FAHFA is far from being completely understood. The first reports show their beneficial effects on glucose homeostasis^36^ and in suppressing inflammation^37^. Furthermore, Kuda *et al*. showed that FAHFA biosynthesis in white adipose tissue is involved in the adaptive Nrf2-regulated antioxidant system^38^. At present, only some aspects of the mechanisms regulating FAHFA expression are known. FAHFA biosynthesis is positively regulated by carbohydrate responsive-element binding protein (ChREBP) and FAHFA levels are elevated also in fasting, probably due to the decreased degradation^35^. In our study, FAHFAs (derived from palmitoleic, palmitolinoleic, linoleic, and linolenic fatty acyl moieties and oleic, linoleic, linolenic, icosaenoic and icosatrienoic hydroxylated fatty acid moieties) were significantly elevated in the liver in both PNIL and PNILOV groups. Our study design does not allow identifying the mechanisms contributing to the PN-associated FAHFA elevation but we may speculate that it is the response to chronic oxidative stress or the sub-optimal saturation of energy needs. Interestingly, albeit the parenteral route of feeding was always associated with a dramatic increase in FAHFA content, the inclusion of ω-3 PUFA resulted in the significant reduction of FAHFA content in the liver. We have not sufficient data to speculate about the biological meaning of this observation but available information indicates that FAHFA are potent lipokines involved in metabolic homeostasis regulation and further research is needed to unravel their role in PN-associated metabolic adaptations.

Due to the high number of double bonds, n-3 PUFA may be more susceptible to lipid peroxidation and may increase the risk of oxidative stress^39^. The expression of enzymes involved in xenobiotic or glutathione metabolism (HSD11B1, FMO1/5, CYP3A9, CYP2C11, GST isoforms) was significantly attenuated in PNIL and PNILOV groups, the effect being more pronounced in PNILOV. All reactions catalyzed by these enzymes are associated with electron transfer and potentially may be the source of reactive oxygen species. The main function of GSTs is to conjugate electrophilic compounds with glutathione, thereby enabling their excretion^40^ but its activity is associated with the depletion of glutathione. The increased intake of PUFA prone to lipid peroxidation may enhance the risk of oxidative stress. Therefore, the decreased activity of these pathways may represent an adaptive mechanism to prevent some PUFA-mediated deteriorative effects such as ROS production. On the other hand, it may negatively affect the effectivity of detoxification.

The published data support both pro- and antioxidant effect of ω-3 PUFA in the context of parenteral nutrition. Human studies are based mostly on the analysis of plasma lipids because of the inaccessibility of liver tissue. In animal studies, two authors confirmed the anti-oxidative effect of fish oil in mice^41^ or rats subjected to intestinal ischemia^42^. In contrast, Lavoie *et al*. described the increased oxidative stress in the lung tissue of newborn guinea pigs administered ω-3 PUFA (SMOFlipid) compared with ω-6 PUFA (Intralipid)^43^. In our study, we did not find the elevated MDA content, the marker of lipoperoxidation, in any group. Nevertheless, we identified early markers of oxidative stress, i.e. the elevated mRNA and protein content of Nrf-regulated genes Hmox1, Nqo1, and Gclc in the liver of all parenterally fed animals, regardless of the type of the lipid emulsion. The antioxidant capacity of liver homogenate was significantly decreased in PNIL (ω-6; PN) but normal in the PNILOV group (ω-6/ω-3; PN). These findings suggest that PN itself is associated with the pro-oxidant status in the liver but ω-3 PUFA protect the anti-oxidative defence capacity. Our study is limited by the short time of the treatment which is insufficient for the full development of the oxidative stress-related injury. Further studies are needed to fully explain the role of ω-3 PUFA in the PN-associated liver oxidative stress.

Cholestasis, i.e. the intrahepatic accumulation of bile acids, is one of the most common metabolic problems associated with PN especially in infants^44^. Lipophilic bile acids, which are often increased in PNALD^5^, are known to cause cellular apoptosis. In contrast, hydrophilic bile acids, i.e. UDCA or tUDCA, have a rather protective effect through activation of mitogen-activated protein kinase survival pathway^45^. gCDCA, the most abundant BA in serum and bile in cholestasis^46^, is highly toxic. *In vitro*, rat and human hepatocytes or hepatic cell lines treated with gCDCA in high concentration (≥100 µM) develop severe mitochondrial dysfunction and apoptosis^47–50^. The exposition to lower, physiological concentrations results in the expression of inflammatory mediators^51^ that stimulate the recruitment of hepatic neutrophils that in turn induce the oxidative injury of the liver tissue. Furthermore, the low-level BAs-induced mitochondrial dysfunction may contribute to stress the hepatocytes and worsen the ongoing inflammatory injury^52^. Recent clinical research demonstrated that pure ω-3 PUFA-based lipid emulsions reversed severe cholestasis in infants or adult patients when administered instead of soybean oil emulsion^10,53,54^. Tillman *et al*. reported that EPA and DHA treatment attenuated the CDCA-induced hepatocellular apoptosis in HepG2 cells *in vitro*^5^. Our data confirmed the accumulation of four toxic BAs (gCDCA, DCA, gDCA and LCA) and depletion of protective tUDCA in the liver of animals administered parenterally ω-6 PUFA. In contrast, no such effect was observed when animals were provided ω-3/ω-6 PUFA mixture what supports the hypothesis about the protective effect of ω-3 PUFA.

### Conclusions

In this study, we employed -omics approach to describe characteristic features of lipid, protein and BAs liver profile associated with enteral or parenteral administration of ω-6 PUFA and ω-3/ω-6 PUFA based lipid emulsions and possible implications for the development of PNALD. In general, we found that enteral administration of both lipid emulsions has less impact on the liver than the parenteral route. Compared with parenterally administered Intralipid, PN administration of ω-3 PUFA was associated with 1. increased content of EPA- and DHA-containing lipid species; 2. higher abundance of CYP4A isoenzymes capable of bioactive lipid synthesis and the increased content of their potential products (oxidized EPA and DHA) with potentially anti-inflammatory properties; 3. down-regulation of enzymes involved CYP450 drug metabolism what may represent an adaptive mechanism counteracting the potential negative effects (enhanced ROS production) of FA metabolism; 4. normalized anti-oxidative capacity; 5. physiological BAs spectrum. All these findings may contribute to the explanation of ω-3 PUFA protective effects in the context of PN. In the present state of knowledge, we cannot assess the physiological relevance of the ω-3 PUFA influence on FAHFA liver content.

## Material and Methods

### Animals and experimental design

Male Wistar rats (Charles River, initial weight 300–325 g) were kept in a temperature-controlled environment under a 12 h light/dark cycle. After the acclimatization period, all animals underwent the same operation procedure. The right jugular vein was cannulated with a Dow Corning Silastic drainage catheter (0.037 inches) as previously described^55^. The catheter was flushed daily with TauroLock HEP-100 (TauroPharm GmbH, Waldbüttelbrunn, Germany). After the operation, the rats were housed individually and connected to a perfusion apparatus (Instech, PA, USA), which allows free movement. For the next 48 hours, the rats were given free access to a standard chow diet (SD, SEMED) and provided Plasmalyte (BAXTER Czech, Prague, CZ) via the catheter at increasing rates (initial rate: 1 ml/hr; goal rate: 4 ml/hr) in order to adapt to the increasing fluid load. Two days after the operation, the rats were randomly divided into five groups as follows: Control (Plasmalyte *i.v*. granules); ENIL (Plasmalyte i.v. Intralipid *per os*, granules); ENILOV (Plasmalyte i.v. Intralipid + Omegaven *per os*, granules); PNIL (nutrition mixture with Intralipid *i.v*.); PNILOV (nutrition mixture with Intralipid + Omegaven *i.v*.) (Supplementary Table 5). The composition of the *i.v*. nutrition mixture is described in Supplementary Table 6. The infusion was applied daily for 10 hrs (CTRL, ENIL, ENILOV, PNIL) or 11 hrs (PNILOV) in the light phase for 12 consecutive days. In ENIL and ENILOV groups, the lipid emulsion was administered three times a day by gavage. Each animal was provided the same amount of energy (60 kcal. day^-1^) and had free access to water. All experiments were performed in accordance with the Animal Protection Law of the Czech Republic 311/1997 in compliance with the Principles of Laboratory Animal Care (NIH Guide for the Care and Use of Laboratory Animals, 8^th^ edition, 2013) and approved by the Ethical Committee of the Ministry of Health, CR (approval no. 53/2014).

### Histological evaluation

Liver tissue samples were fixed in 4% paraformaldehyde, embedded in paraffin blocks and routinely processed. Sections cut at 4–6 µm were stained with hematoxylin/eosin and examined with an Olympus BX41 light microscope.

### Biochemical analyses

Biochemical analyses were performed using commercially available kits (Roche Diagnostics, Basel, Switzerland; ALT-(ALAT/GPT) cat.no.: 10851132; AST-(ASAT/GOT) catno.: 10851124; TG-Triglycerides GPO-PAP cat.no.: 11730711; Bilirubin – BLT3 cat.no.: 05795320) on Hitachi analyzer 902. Statistical analyses for biochemical assays were performed using Graph PadPrism version 5.03 for Windows. Results were expressed as means ± standard deviation (SD). Kruskal-Wallis test with Bonferroni correction for multiple comparisons was performed to determine significant differences between the groups at a significance level of p < 0.05.

### Determination of malondialdehyde concentration

The total malondialdehyde (MDA) content in liver homogenate was determined using stable isotope dilution assay based on liquid chromatography-tandem mass spectrometry. An internal standard of 1,3-dideuteromalondialdehyde (MDA-D2) stock solution was prepared by acid hydrolysis of 1,3-D2-1,1,3,3-tetraethoxypropane (Cambridge Isotope Laboratories, Tewksbury MA, USA). 100 µl of washed erythrocytes was mixed with 10 µl of diluted internal standard MDA-D2 (10 µM) and lysed with four volumes of cold distilled water in a refrigerator at 4 °C for 15 min; the cell debris was removed by centrifugation. The concentration of hemoglobin was measured at 540 nm using an extinction coefficient ${E}_{540}^{1 \% }$ = 8.5. For plasma samples, 100 µl of EDTA plasma was mixed with 10 µl of internal standard MDA-D2 (10 µM) The supernatant was hydrolyzed with NaOH (1 M final concentration) for 30 min at 60 °C. To the hydrolysate, 3 M HClO_4_ was added for protein precipitation and samples were centrifuged. The supernatant was derivatized by 5 mM 2,4-dinitrophenylhydrazine (DNPH). The reaction mixture was centrifuged and 20 µl was injected onto HPLC column Nucleosil C18 ec (125 × 3 mm, 5 μm) (Macherey-Nagel, Düren, Germany) at 40 °C using isocratic mobile phase composed of 0.1% of formic acid in 50% acetonitrile (v/v), flow rate 400 μl/min.

The HPLC system was connected to the mass spectrometer QTRAP 4000 (Sciex, Prague, Czech Republic). MDA and MDA-D2 DNPH derivatives (MDA-DNPH and MDA-D2-DNPH) were detected in positive multiple reaction monitoring (MRM) mode. MDA-DNPH was monitored at *m/z* 235→189 and MDA-D2-DNPH at *m/z* 237→191. MDA and MDA-D2 DNPH derivatives eluted at 3.00 min. Analyst v.1.6 from SCIEX was used for the acquisition and analysis of data.

### Real-time quantitative PCR

Liver samples were dissected and immediately frozen in liquid nitrogen. Total RNA was extracted using the Qiagen Mini RNeasy isolation kit (Qiagen, Hilden, Germany). A DNAase step was included to avoid possible DNA contamination. The integrity was detected on the fragment analyzer Automated CE System (Advanced Analytical Technologies, Inc., Ames, USA). The concentration of total RNA was measured on Qubit (Thermofisher Scientific, USA). A standard amount of total RNA (700 ng) was used to synthesize the first-strand cDNA with the High Capacity RNA-to-cDNA Kit (Applied Biosystems, Foster City, CA, USA). The RT-PCR amplification mixture (20 ul) contained 1 ul template cDNA, 5xHOT FIREPol Eva Green qPCR SuperMix (Solis BioDyne, Tartu, Estonia) and 400 nM (10 pmol/reaction) sense and antisense primer. The reaction was run on the ViiA 7 Real-Time PCR System (ThermoFisher Scientific, USA). Results were analyzed using ViiA software v 1.1 (ThermoFisher Scientific, USA). The expression of genes of interest was normalized to the housekeeper gene (B2M and Cyc1) and calculated using the ΔΔCt method.

### Primer design

Primers were based upon known rat sequences available from the GeneBank Graphics database https://www.ncbi.nlm.nih.gov. List of primers is shown in Supplementary Table 7.

### Lipidomics analysis

The liver extracts were prepared by a previously described method relying on MTBE:MeOH (methyl tert-butyl ether: methanol) extraction^56^ with the modification of resuspending the dried extract in a mixture of isopropyl alcohol:MeOH:H_2_O (65:30:5,v/v/v), the full preparation is described in Supplementary Methods. For the lipidomic analysis, U-HPLC (Infinity 1290, Agilent) coupled to a high-resolution mass spectrometer with a hyphenated quadrupole time-of-flight mass analyzer (6560 Ion Mobility Q-TOF LC/MS; Agilent) with the Agilent Jet Stream (AJS) electrospray (ESI) source were employed. Mass spectrometer acquired data in the m/z range of 100–1200 in MS1 mode for all the samples and a repeatedly (every 10 samples) injected QC sample. Fragmentation experiments for lipid identification were carried out on the QC sample using ddMS2 acquisition mode. The collision energy was set to 10, 20 and 40 eV. Chromatographic separation was based on 150 mm BEH C18 reverse-phase column (details in supplemental) with a mobile phase gradient of A: ACN:H2O 60:40) and B: 2-propanol/ACN (90:10) using ammonium formate and formic acid (ESI+) or ammonium acetate and acetic acid (ESI-) as additives.

### Lipid identification and statistical analyses

The mass spectrometry data was processed LipidMatch suite^57^ which uses MZmine 2 for feature extraction and an R script for lipid identification based on in-silico fragmentation databases. At least a class-specific fragment was required for lipid identification. One item, FAHFA (18:2/18:2)_FAHFA(16:1/20:3), was not identified unequivocally. Due to the number of isomers the separation technique does not allow to separate some of them properly, the fragmentation spectrum contains fatty acyl ions confirming both lipid identities.

Lipidomics data processing was performed in both web-based and R based MetaboAnalyst (metaboanalyst.ca) packages followed by SIMCA (Umetrics) and subjected to two-dimensional hierarchical cluster analysis (HCA). A custom-built R script based on the MetaboAnalystR package was used to filter out features based on their univariate statistics. In MetaboAnalyst, sum normalization, log transformation, and Pareto scaling were used for signal processing. Statistically insignificant compounds were filtered out if they did not meet the criteria of a t-test or ANOVA p-value < 0.01 (FDR adjusted). These filtered data matrices were the first sum normalized in MS Excel and then loaded to SIMCA, where statistical models were built. When OPLS-DA (Orthogonal PLS-DA) models in SIMCA logarithmic transformation and Pareto scaling was used. Fragmentation spectra of the VIP score based significant compounds were double-checked both against the in-silico fragmentation library and against METLIN and LIPIDMAPS databases for their identities to be confirmed. Profiles of oxidized forms of free fatty acids were obtained by feeding a database with their respective molecular formulas to MassHunter Profinder, which scans the LC-HRMS data for respective features, which satisfy molecular formula requirements. These requirements were set as m/z value of 5 ppm, high confidence of isotopic pattern distribution and isotope spacing within 10 ppm. The features with a score of less than 80 were discarded.

### Proteomic analysis

The tissue was lysed in the detergent-containing buffer, cysteines were reduced, blocked and samples were digested with trypsin^58^. The samples were injected on nanoLC coupled with Orbitrap Fusion™ Tribrid™ (Thermo Scientific, USA, San Jose) mass spectrometer. Data were collected during a two-hour gradient. For protein identification and quantification were used MaxQuant software. The search was performed with the Rattus Norvegicus UniProt proteome database from 11/2018 (29965 entries). Gene names are listed according to HGNC guidelines (https://www.gennames.org). The detailed workflow is present in Supplementary Methods.

### Bile acid analysis

The BAs were determined in methanol extract of lyophilized liver tissue. Liquid chromatography separation was performed using 1290 Infinity LC (Agilent Technologies) followed by mass spectrometry using 6550 iFunnel LCQ- TOF-MS (Agilent Technologies) equipped with a Dual AJS ESI probe in negative-ion mode. System control and data acquisition were performed by Agilent MassHunter Quadrupole Time of Flight Acquisition Software (B.06) with Qualitative Analysis (B.07 SP2) Software. The detailed workflow is present in Supplementary Methods.

## Supplementary information

Supplementary Methods

Supplementary Figures

Supplementary Tables
