# Supplementary material for: The effect of ω-3 polyunsaturated fatty acids on the liver lipidome, proteome and bile acid profile: parenteral versus enteral administration
Source: Sci Rep. 2019 Dec 13;9:19097. doi: 10.1038/s41598-019-54225-8 (PMC6910966; doi:10.1038/s41598-019-54225-8)
Supplement: Supplementary file 1 — Supplementary Methods [file 41598_2019_54225_MOESM1_ESM.pdf]

The effect of ω-3 polyunsaturated fatty acids on the liver lipidome, proteome and bile acid profile: parenteral *versus* enteral administration

Kamila Bechynska, Nikola Daskova, Nikola Vrzackova, Karel Harant, Marie Heczková, Katerina Podzimkova, Miriam Bratova, Helena Dankova, Zuzana Berkova, Vit Kosek, Jaroslav Zelenka, Jana Hajslova, Radislav Sedlacek, Jiri Suttnar, Alzbeta Hlavackova, Lenka Bartonova, Monika Cahova

Supplementary methods

Lipidomics analysis

The liver extract was prepared as previously described (Wang 2016). Liver tissue (10 mg/400 µl) was homogenized in cold MetOH : H_2O (3:1, v/v) on Ultra Turax ( ). After homogenization, 1 ml methyl tert-butyl ether (MTBE) was added and the mixture was shaken for 15 min. Subsequently, 300 µl of deionized water was added to form a two-phase system. After vortexing for 30 s, the obtained mixture was left to stand for 10 min at 4°C. Liver lipid extracts were then deproteinized by centrifugation at 10,000 rpm (10,621 ***g***) for 10 min at 4°C. The resultant supernatants were lyophilized and stored in an −80°C freezer if needed. The freeze-dried lipid residues were resuspended in isopropylalcohol/methanol/deionized water (65:30:5, v/v/v) and used for subsequent analysis.

For the lipidomic analysis, U-HPLC (Infinity 1290, Agilent) coupled to a high-resolution mass spectrometer with a hyphenated quadrupole time-of-flight mass analyzer (6560 Ion Mobility Q-TOF LC/MS; Agilent) with the Agilent Jet Stream (AJS) electrospray (ESI) source were employed.

The mass analyzer was operated at following conditions at both ionization modes: Gas temperature 350 °C, Drying Gas 12 L/min, Nebulizer pressure 40 psig, Sheath gas temperature 350 °C, Sheath gas flow 11 l/min, Nozzle voltage 250 V, fragmentor voltage 380 V, octopole radiofrequency voltage 750 V. The capillary voltage was 3000 V in ESI+, 4000 V in ESI-. For all samples, the data were acquired over the m/z range of 100 – 1700 at the rate of 1 spectrum/s. The m/z range was autocorrected on reference masses 121.0509 and 922.0098 for positive mode and 119.0363 and 980.0164 in negative mode. To obtain the fragmentation spectra of lipids, the QC sample was run several times in auto MS/MS mode with collision energies 10, 20 and 40 eV, with acquisition rate of 5 spectra/s for full spectra and 7 spectra/sec for fragmentation spectra. Top 5 ions were chosen for fragmentation with the dynamic exclusion for 0.1 min after fragmentation event.

For lipidomic fingerprinting an Acquity BEH C18 (1.7 µm, 2.1 mm x 150 mm (Waters, USA) was used for chromatographic separation. The chromatographic system used with ESI+ detection was: A – 10 mM ammonium formate and 0.1 % formic acid in acetonitrile:water (60:40, v/v); B – 10 mM ammonium formate and 0.1 % formic acid in 2-propanol:acetonitrile (90:10, v/v). For chromatographic separation of liver detected in ESI- mode, following mobile phases were used: A - 10 mM ammonium acetate and 0.1% acetic acid in acetonitrile:water (60:40); B - 10 mM ammonium acetate and 0.1% acetic acid in 2-propanol:acetonitrile (90:10, v/v). The flow rate was constant at 0.300 $mL \cdot min^{-1}$. The mobile phase gradient is described in Supplementary Table S2. The column temperature was maintained at 60°C, the injection volume was increased to 1 µL in ESI+ mode and 5 µL in ESI- mode. The autosampler was kept at 10 °C.

QC sample was run every 10 samples for system stability assessment. The samples were measured in MS mode, the fragmentation experiments were run on the QC sample at the end of the batch at 10, 20 and 40 eV.

Proteomic analysis

Tissue was lysed in 100mM TEAB (Pierce, 90114) buffer with 2% SDC (Sigma, D750). Protein concentration was determined using BCA protein assay kit (Thermo) and 30 µg of protein per sample was used for MS sample preparation. Cysteines were reduced with 5mM TCEP (Thermo Scientific, 20491) 60°C for 60 min and blocked with 10mM MMTS (Sigma, 64306) for 10 min at room temperature. Samples were digested with trypsin (trypsin/protein ration 1/20) at 37°C overnight. After digestion samples were acidified with TFA to 1% final concentration. SDC was removed by extraction to ethyl acetate (Masuda 2008) and peptides were desalted on Michrom C18 column.

Nano Reversed phase columns (EASY-Spray column, 50 cm x 75 µm ID, PepMap C18, 2 µm particles, 100 Å pore size) were used for LC/MS analysis. Mobile phase buffer A was composed of water and 0.1% formic acid. Mobile phase B was composed of acetonitrile and 0.1% formic acid. Samples were loaded onto the trap column (Acclaim PepMap300, C18, 5 µm, 300 Å Wide Pore, 300 µm x 5 mm, 5 Cartridges) for 4 min at 17.5 µl/min loading buffer was composed of water, 2% acetonitrile and 0.1% trifluoroacetic acid. Peptides were eluted with Mobile phase B gradient from 4% to 35% B in 120 min. Eluting peptide cations were converted to gas-phase ions by electrospray ionization and analyzed on a Thermo Orbitrap Fusion (Q-OT- qIT, Thermo Scientific). Survey scans of peptide precursors from 350 to 1400 m/z were performed at 120K resolution (at 200 m/z) with a 5×10^5 ion count target. Tandem MS was performed by isolation at 1,5 Th with the quadrupole, HCD fragmentation with a normalized collision energy of 30, and rapid scan MS analysis in the ion trap. The MS2 ion count target was set to 10^4 and the max injection time was 35 ms. Only those precursors with charge state 2–6 were sampled for MS2. The dynamic exclusion duration was set to 45 s with a 10 ppm tolerance around the selected precursor and its isotopes. Monoisotopic precursor selection was turned on. The instrument was run in top speed mode with 2s cycles (Herbert 2014).

Protein identification and data analysis

All data were analyzed and quantified with the MaxQuant software (version 1.6.1.0) (Cox 2008). The false discovery rate (FDR) was set to 1% for both proteins and peptides and we specified a minimum peptide length of seven amino acids. The Andromeda search engine was used for the MS/MS spectra search against the *Rattus norvegicus* (downloaded from Uniprot on October 2018, containing 29965 entries). Enzyme specificity was set as C-terminal to Arg and Lys, also allowing cleavage at proline bonds and a maximum of two missed cleavages. Dithiomethylation of cysteine was selected as fixed modification and N- terminal protein acetylation and methionine oxidation as variable modifications. The “match between runs” feature of MaxQuant was used to transfer identifications to other LC-MS/MS runs based on their masses and retention time (maximum deviation 0.7 min) and this was also used in quantification experiments. Quantifications were performed with the label-free algorithm in MaxQuant (Cox 2014).

Bile acid analysis

One milliliter of methanol (LC/MS grade, Honeywell) was added to 50 mg of the lyophilized liver tissue to extract BAs. The samples were sonicated (amplitude 50%, 15 s on, 10 s off, 4 cycles, on ice; Sonoplus HD3100, Bandelin) and then heated to 60°C for 45 minutes in the heat block (Thermo-shaker TS-100, Biosan). After cooling to room temperature, the samples were centrifuged at 1600 g for 10 min at 15°C and the supernatants were collected. To the precipitates, the same volume of methanol as in the first extraction step was added and mixed vigorously by vortex for 1 min. The samples were centrifuged at 20000 g for 10 min at 2°C and supernatants were collected. This extraction step was repeated once more. To the pooled extracts, a mix of labeled BAs (10 nM) was added as internal standard. Samples were centrifuged at 20000 g for 10 min at 2°C. For biochemical analysis, 50 µl of pooled extracts in methanol (described above) were used and diluted 1:1 with dH_20.
Liquid chromatography (LC) separation was performed using 1290 Infinity LC (Agilent Technologies) with a gradient elution from a Pinnacle DB C18 column (1.9 µm, 100 mm x 2.1 mm; Restek) and maintained at 55°C and a flow rate of 300 µl/min. The autosampler was kept at 10°C. The sample injection volume was 2 µl. Solvent A was water (LC/MS grade, VWR International) containing 10 mM NH_4F. Solvent B was 100 % methanol (LC/MS grade, Honeywell). The gradient was changed linearly and started with 40% solvent B at 0 min with increasing to 57% within 3.5 min, then 59% until 9.5 min, then 70% until 10 min, then 72% until14min, 76% until 16 min and at last 95% for 9 minutes with 5 min post run for column equilibration with 40% methanol. The column eluent was introduced into the MS.

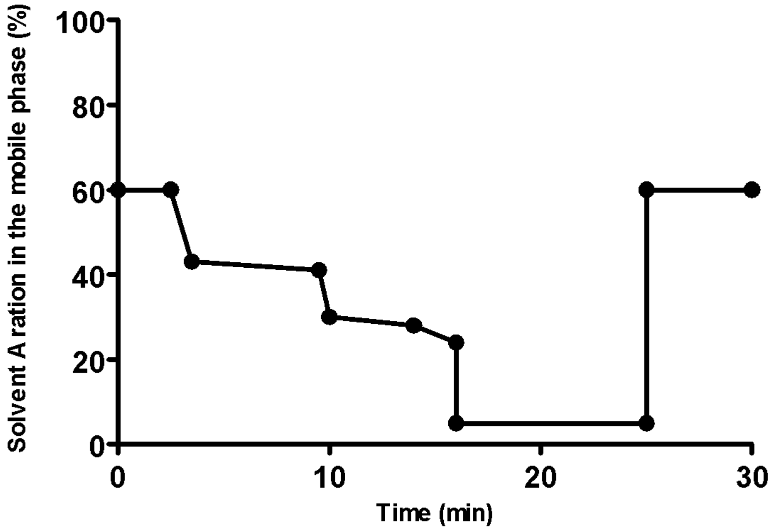

MS analysis was performed using 6550 iFunnel Q-TOF LC/MS (Agilent Technologies) equipped with a Dual AJS ESI probe in negative-ion mode. A capillary voltage of -3500 V, a gas temperature of 120°C, and a sheath gas temperature of 350°C were used. The nozzle voltage was 500 V, the drying gas and sheath gas flow were 14 l/min and 11 l/min, respectively.
The m/z values and retention time values of BAs were investigated in a preliminary experiment within the same experimental setup by three successive analyses of single standard mixture samples.

System control and data acquisition were performed by Agilent MassHunter Quadrupole Time of Flight Acquisition Software (B.06) with Qualitative Analysis (B.07 SP2) Software. Data were analyzed using Find by Formula and database search software functions. Acceptance criteria included a match to retention time (within 0.02 minutes), isotope spacing and abundance, accurate mass (within 5 ppm), MS spectral database matching, and overall score (>75). Concentrations of individual BAs were calculated from the peak areas detected in chromatogram relative to the internal standard, each BA with its own deuterium or C13 labeled BA (Cambridge Isotopes).

References

Masuda, T., Tomita, M., Ishihama, Y., (2008). Phase Transfer Surfactant-Aided Trypsin Digestion for Membrane Proteome Analysis. Journal of Proteome Research 7:731-740

Hebert, A. S., A. L. Richards, D. J. Bailey, A. Ulbrich, E. E. Coughlin, M. S. Westphall and J. J. Coon (2014). "The one hour yeast proteome." Mol Cell Proteomics 13(1): 339-347.

Cox, J., M. Y. Hein, C. A. Luber, I. Paron, N. Nagaraj and M. Mann (2014). "Accurate proteome-wide label-free quantification by delayed normalization and maximal peptide ratio extraction, termed MaxLFQ." Mol Cell Proteomics **13**(9): 2513-2526.

Cox, J. and M. Mann (2008). "MaxQuant enables high peptide identification rates, individualized p.p.b.-range mass accuracies and proteome-wide protein quantification." Nat Biotechnol **26**(12): 1367-1372.
