# Supplementary material for: The effect of ω-3 polyunsaturated fatty acids on the liver lipidome, proteome and bile acid profile: parenteral versus enteral administration
Source: Sci Rep. 2019 Dec 13;9:19097. doi: 10.1038/s41598-019-54225-8 (PMC6910966; doi:10.1038/s41598-019-54225-8)
Supplement: Supplementary file 2 — Supplementary Figures [file 41598_2019_54225_MOESM2_ESM.pdf]

The effect of ω-3 polyunsaturated fatty acids on the liver lipidome, proteome and bile acid profile: parenteral *versus* enteral administration

Kamila Bechynska, Nikola Daskova, Nikola Vrzackova, Karel Harant, Marie Heczková, Katerina Podzimkova, Miriam Bratova, Helena Dankova, Zuzana Berkova, Vit Kosek, Jaroslav Zelenka, Jana Hajslova, Radislav Sedlacek, Jiri Suttnar, Alzbeta Hlavackova, Lenka Bartonova, Monika Cahova

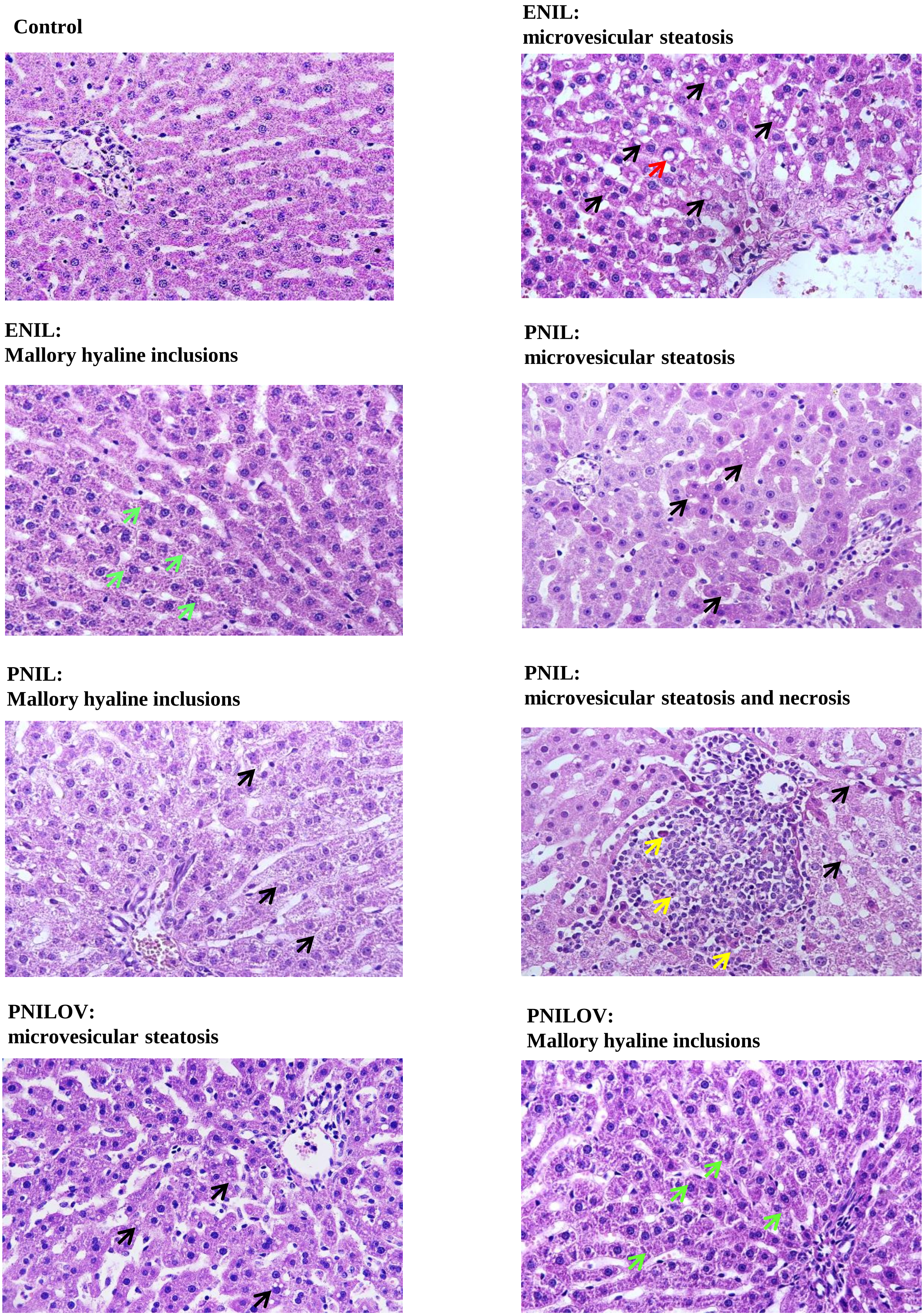

Supplementary Figure 1 *Histological evaluation of the liver*. Black arrows: lipid droplets; red arrows: glycogen inclusion; green arrows: Mallory hyaline inclusions; yellow arrows: apoptotic bodies. Magnification 600x.

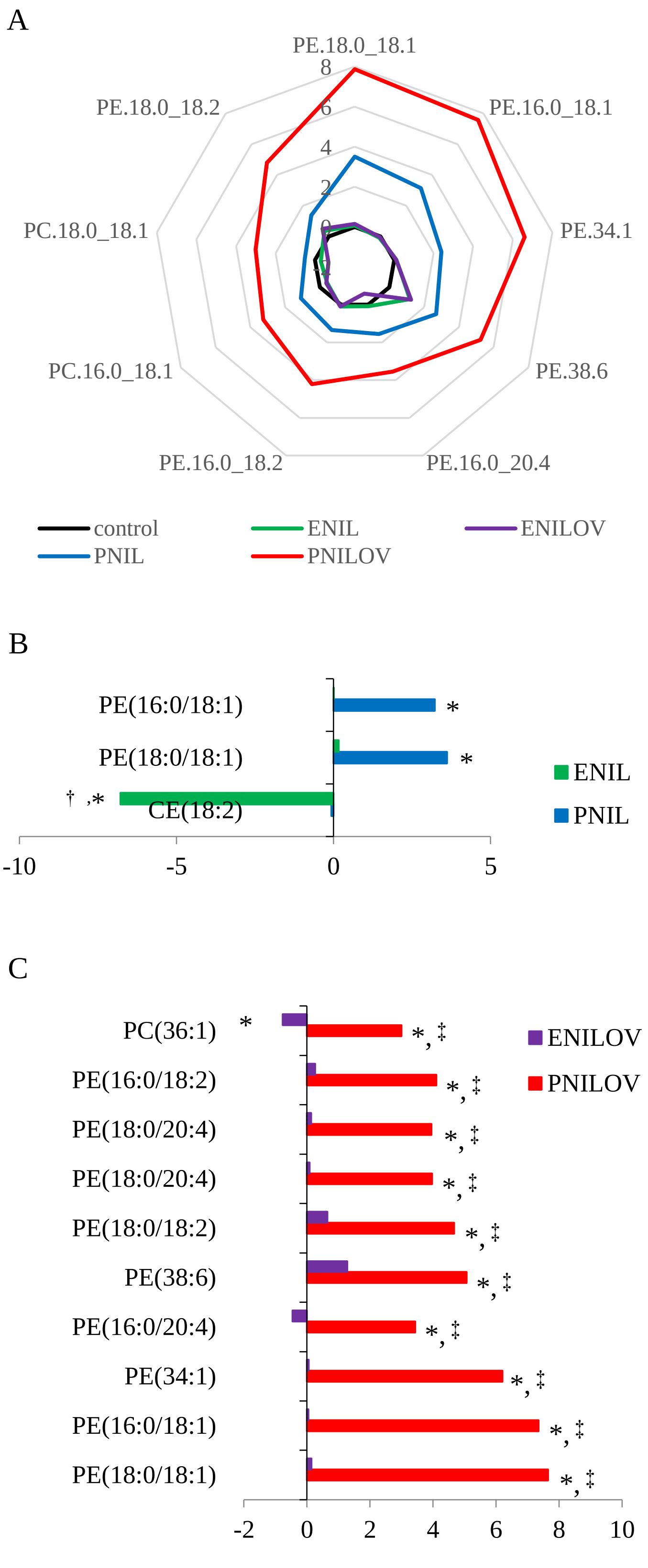

Figure S2 *Effect of the route of administration on the relative content of selected lipid species in serum*. A: all groups; B: PN vs EN: Intralipid; C: PN vs EN: Intralipid + Omegaven
Data are expressed as log(2) fold change over median control value. * p < 0.05 vs control; † p < 0.05 vs PNIL; ‡ < 0.05 vs ENILOV.

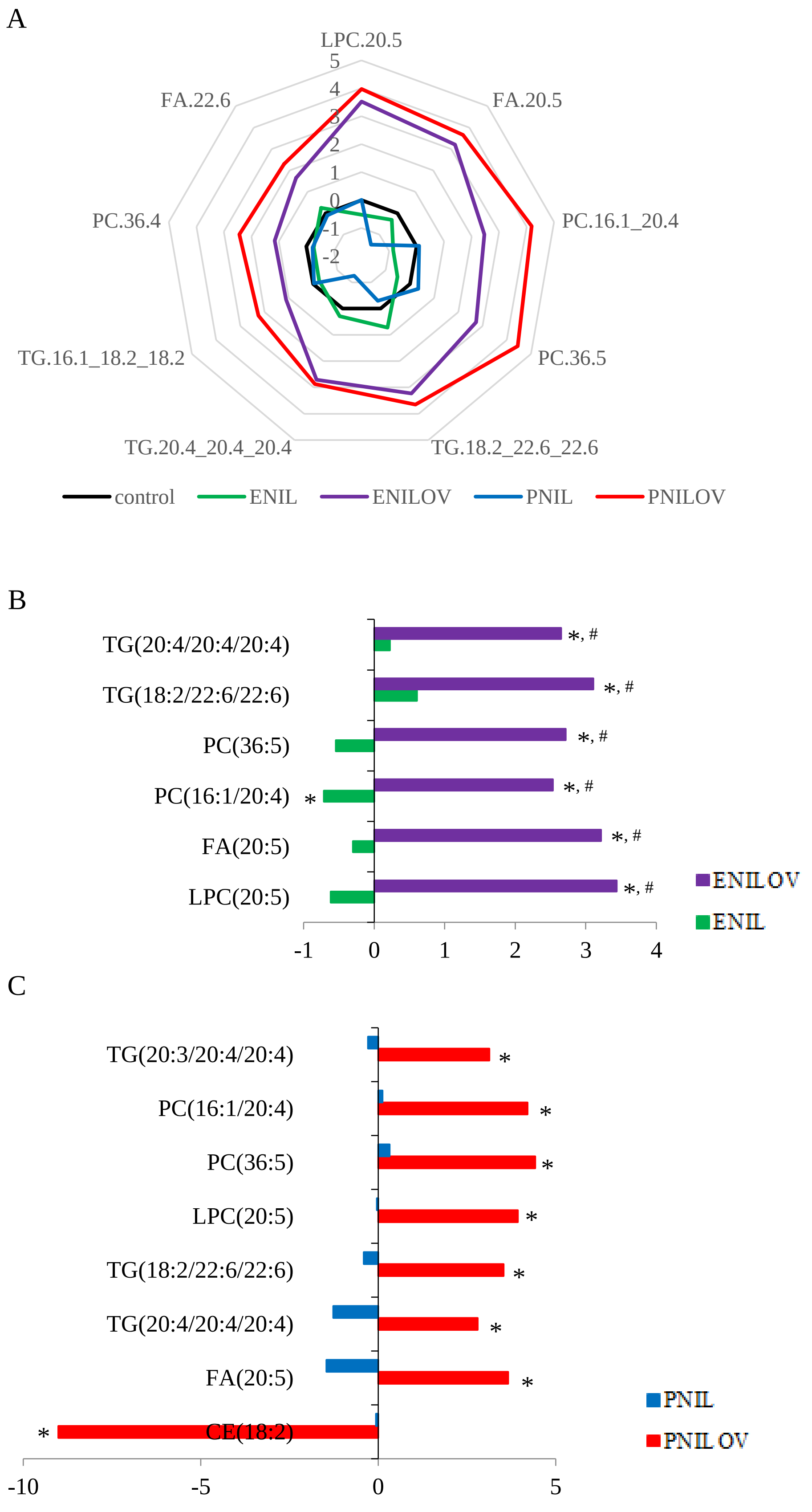

Figure S3 *Effect of the composition of lipid emulsions on the relative content of selected lipid species in serum*. A: all groups; B: IL vs ILOV: enteral application; C: IL vs ILOV: parenteral application. Data are expressed as log(2) fold change over median control value. * $p < 0.05$ vs control; $^{\#}$ $p < 0.05$ vs ENIL; $^{\&}$ $p < 0.05$ vs PNILOV

Phosphatidylcholines

A

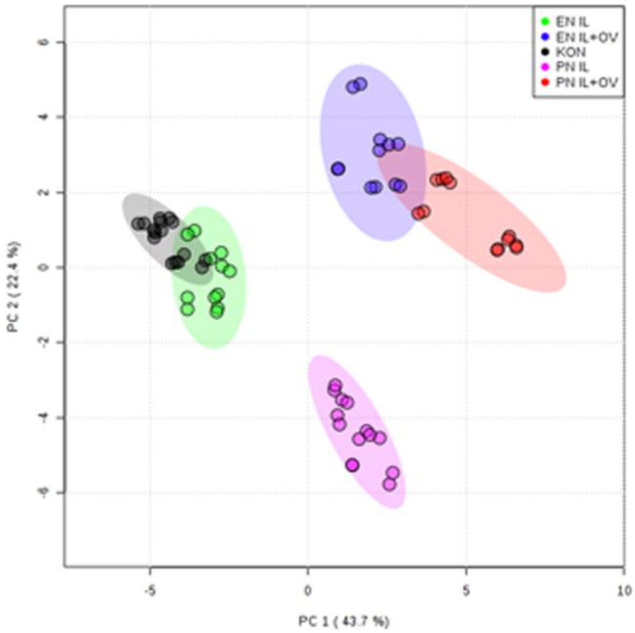

B

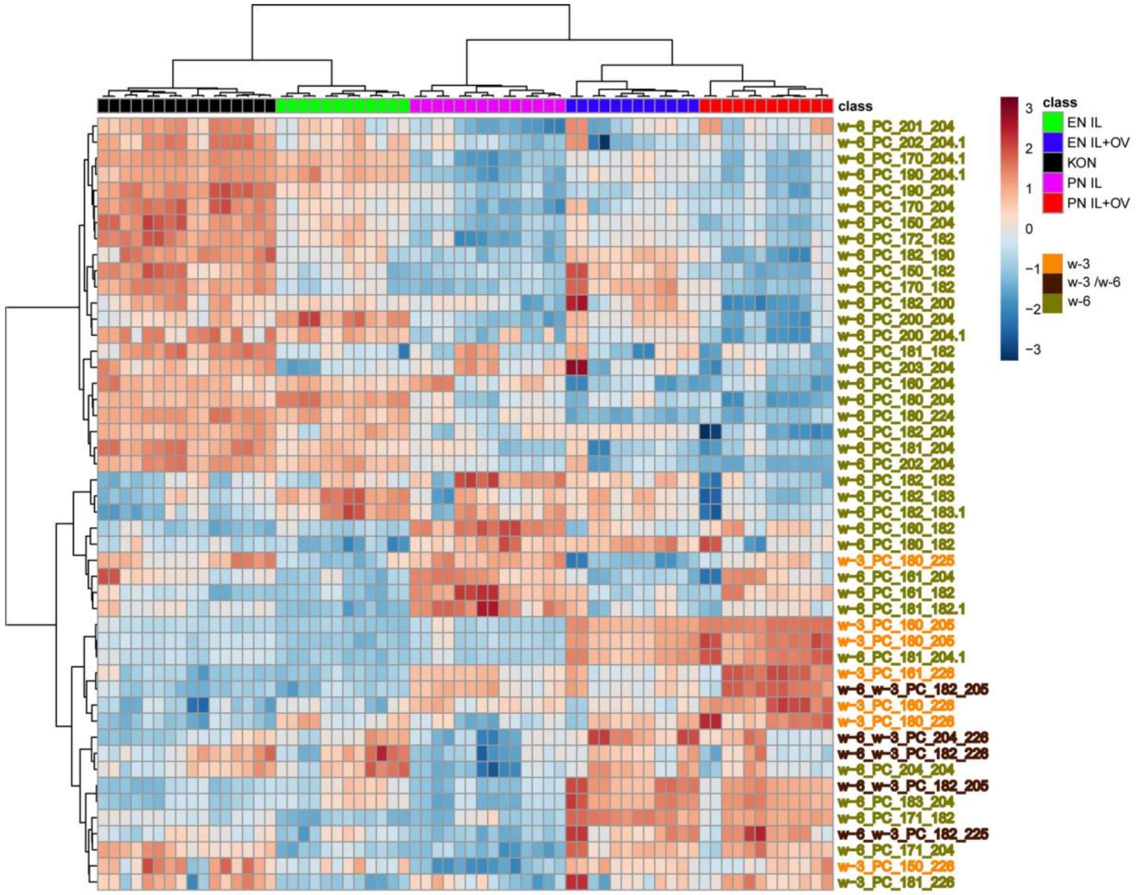

Figure S4 *The distribution of phosphatidylcholines containing ω-3 and/or ω-6 fatty acids in experimental groups.* A: PCA score plot. Each sample was determined in a doublet. B: Heatmap with the clustering dendrogram of samples. Samples are colored according to the experimental groups, individual compounds are colored according to the presence of ω-3 FAs, ω-6 FAs or both.

Phosphatidylethanolamines

A

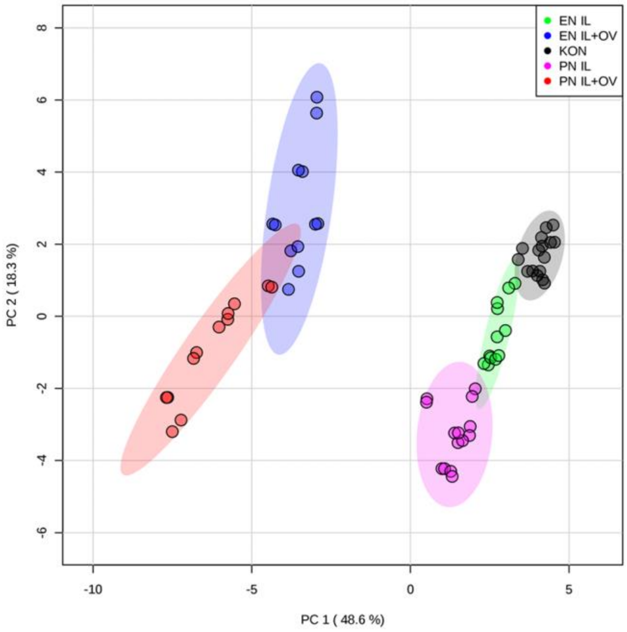

B

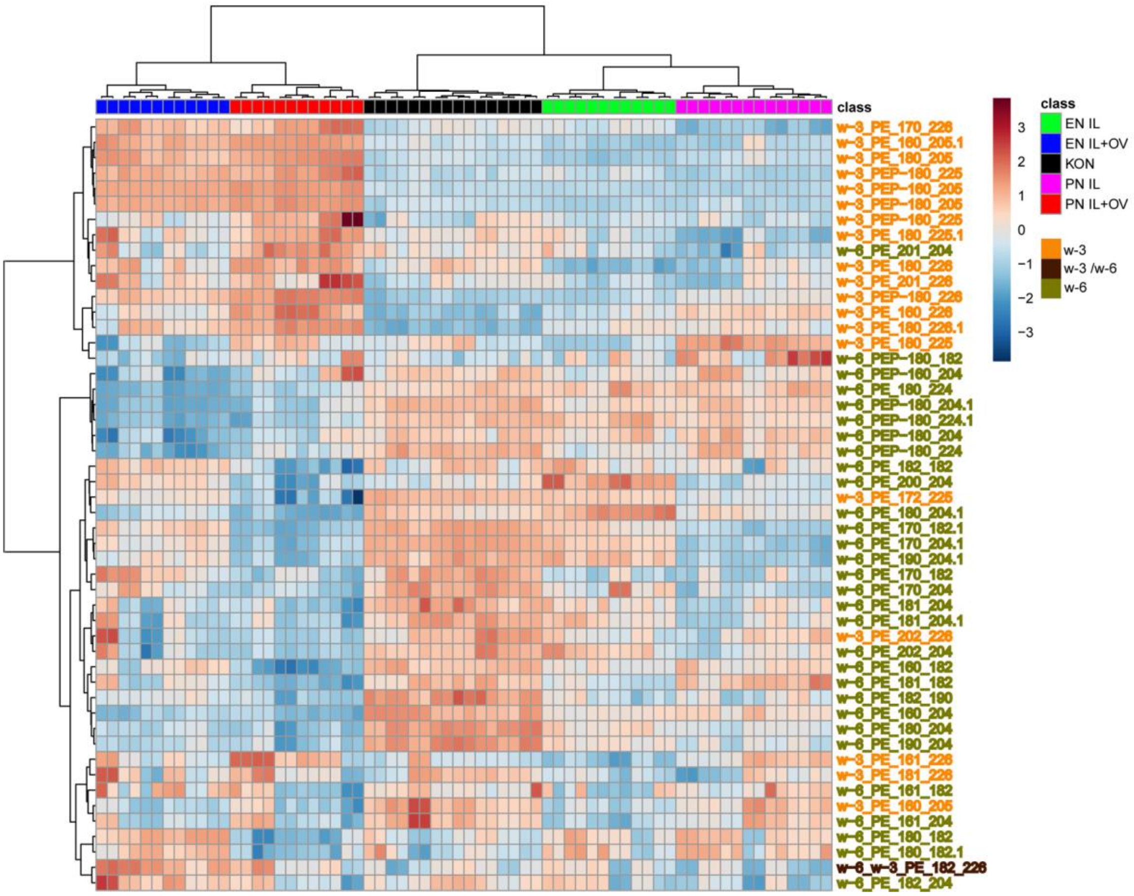

Figure S5 *The distribution of phosphatidylethanolamines containing ω-3 and/or ω-6 fatty acids in experimental groups.* A: PCA score plot. Each sample was determined in a doublet. B: Heatmap with the clustering dendrogram of samples. Samples are colored according to the experimental groups, individual compounds are colored according to the presence of ω-3 FAs, ω-6 FAs or both.

Diacylglycerols

A

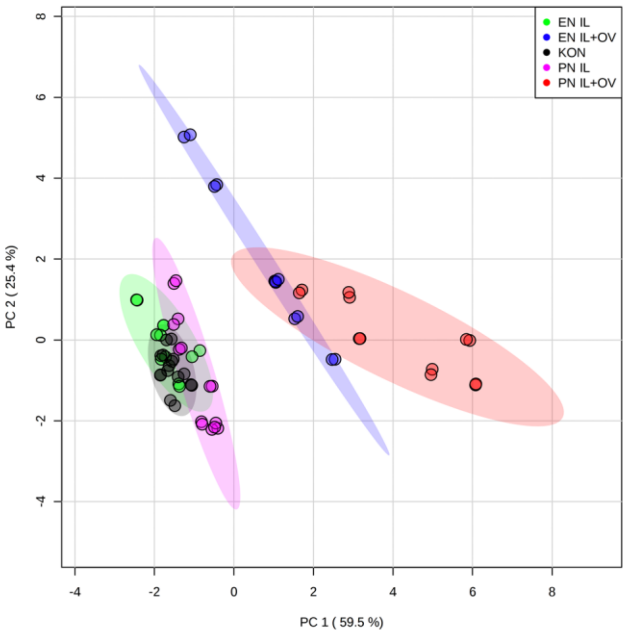

B

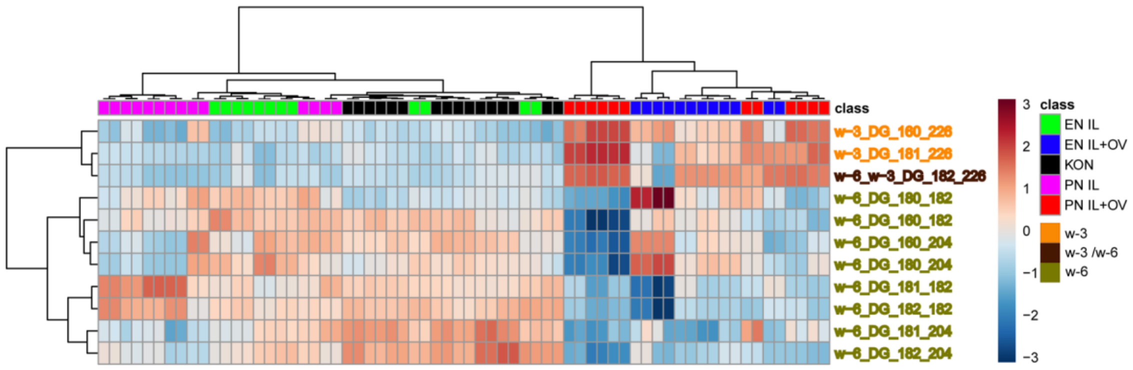

Figure S6 *The distribution of diacylglycerols containing ω-3 and/or ω-6 fatty acids in experimental groups.* A: PCA score plot. Each sample was determined in a doublet. B: Heatmap with the clustering dendrogram of samples. Samples are colored according to the experimental groups, individual compounds are colored according to the presence of ω-3 FAs, ω-6 FAs or both.

Lysophospholipids

A

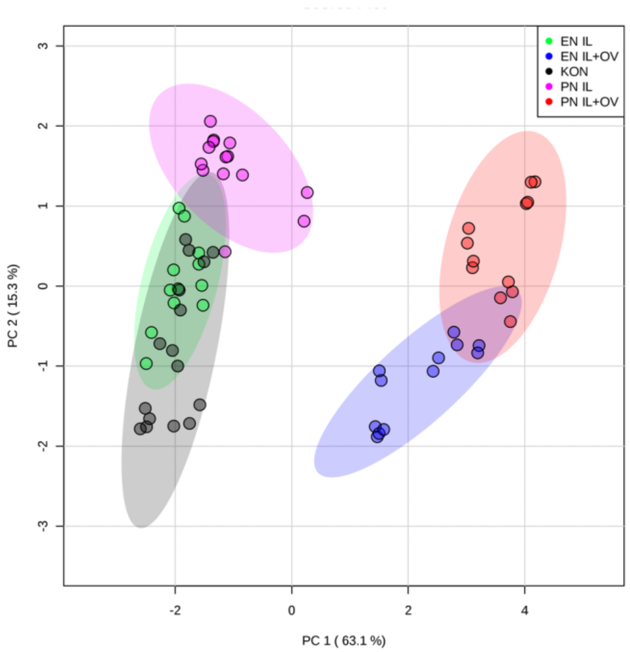

B

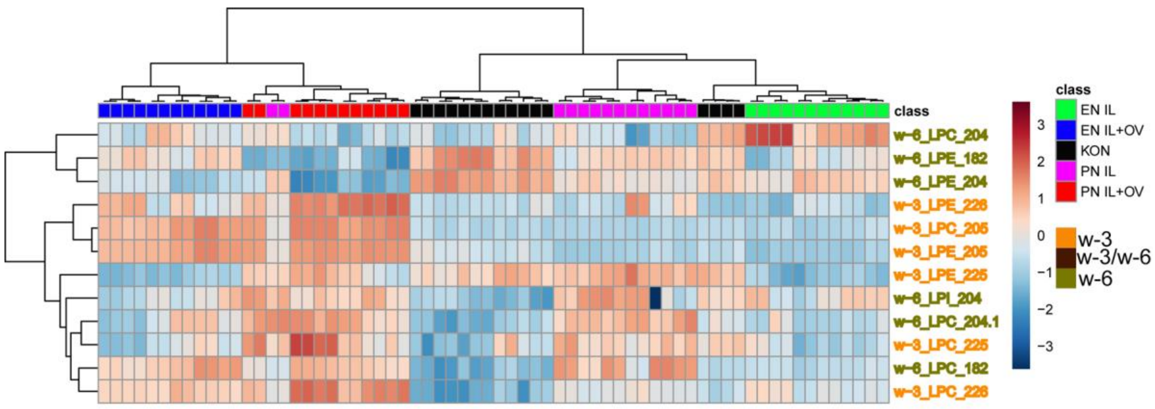

Figure S7 *The distribution of lysophospholipids containing ω-3 and/or ω-6 fatty acids in experimental groups.* A: PCA score plot. Each sample was determined in a doublet. B: Heatmap with the clustering dendrogram of samples. Samples are colored according to the experimental groups, individual compounds are colored according to the presence of ω-3 FAs, ω-6 FAs or both.

Free fatty acids

A

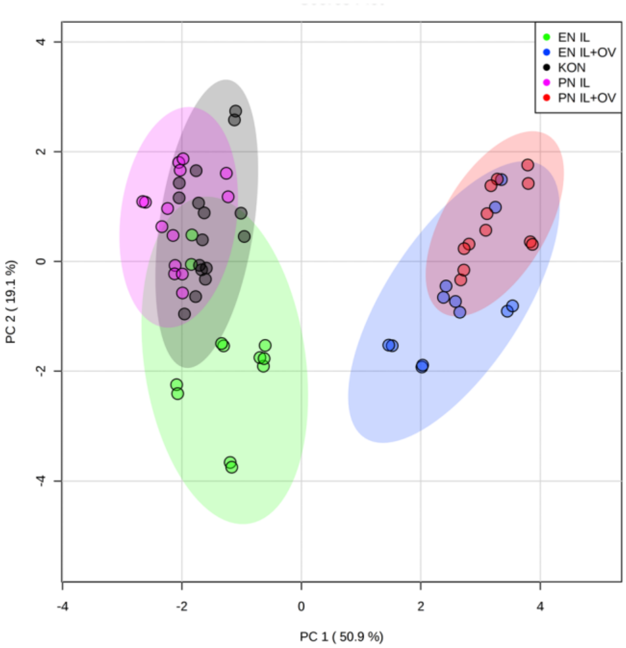

B

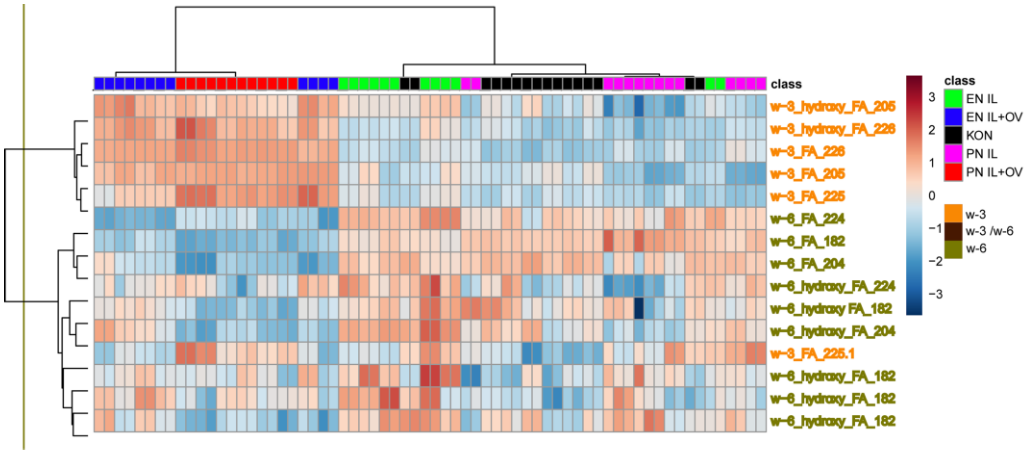

Figure S8 *The distribution of fatty acids in experimental groups (liver).* A: PCA score plot. Each sample was determined in a doublet. B: Heatmap with the clustering dendrogram of samples. Samples are colored according to the experimental groups, individual compounds are colored according to the presence of ω-3 FAs, ω-6 FAs or both.

Triacylglycerols

A

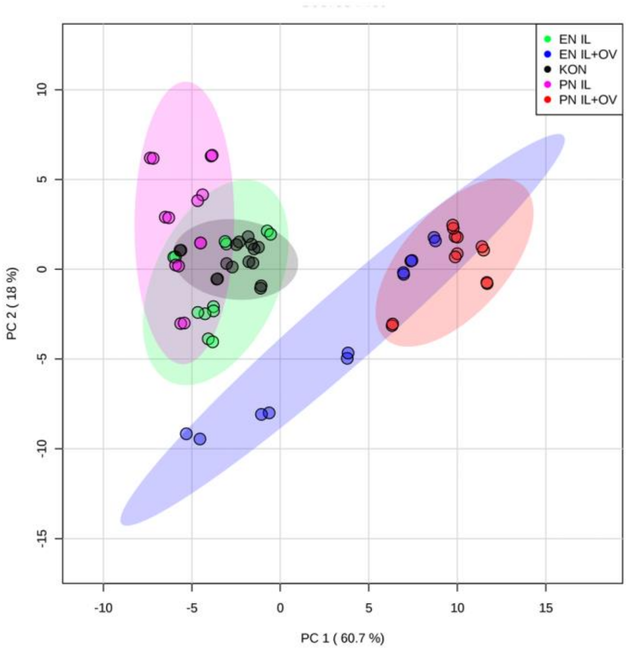

B

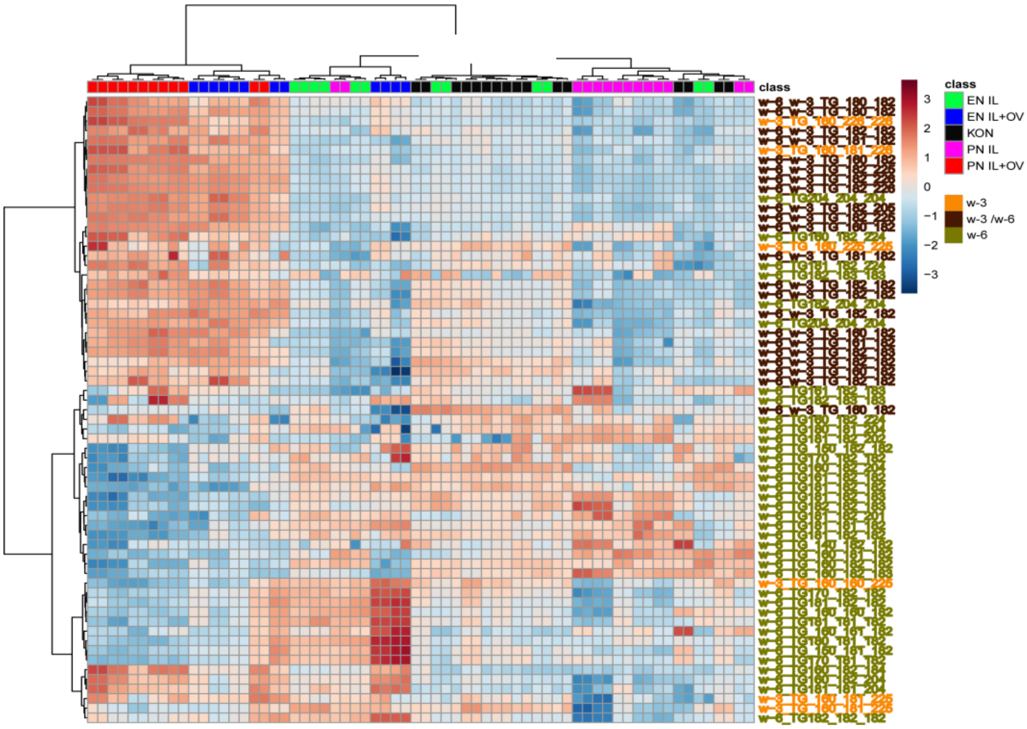

Figure S9 *The distribution of triacylglycerols containing ω-3 and/or ω-6 fatty acids in experimental groups.* A: PCA score plot. Each sample was determined in a doublet. B: Heatmap with the clustering dendrogram of samples. Samples are colored according to the experimental groups, individual compounds are colored according to the presence of ω-3 FAs, ω-6 FAs or both.
