# Supplementary material for: The effect of ω-3 polyunsaturated fatty acids on the liver lipidome, proteome and bile acid profile: parenteral versus enteral administration
Source: Sci Rep. 2019 Dec 13;9:19097. doi: 10.1038/s41598-019-54225-8 (PMC6910966; doi:10.1038/s41598-019-54225-8)
Supplement: Supplementary file 3 — Supplementary Tables [file 41598_2019_54225_MOESM3_ESM.pdf]

The effect of ω-3 polyunsaturated fatty acids on the liver lipidome, proteome and bile acid profile: parenteral *versus* enteral administration

Kamila Bechynska, Nikola Daskova, Nikola Vrzackova[1], Karel Harant, Marie Heczkova, Katerina Podzimkova, Miriam Bratova, Helena Dankova, Zuzana Berkova, Vit Kosek, Jaroslav Zelenka, Jana Hajslova,
Radislav Sedlacek, Jiri Suttnar, Alzbeta Hlavackova, Lenka Bartonova, Monika Cahova

Supplementary Table 1 *Serum VIP lipids*

PNIL vs control		ENIL vs control		PNILOV vs control		ENILOV vs control		PNIL vs ENIL		PNILOV vs ENILOV		PNIL vs PNILOV		ENIL vs ENILOV	
Lipid name	VIP score		VIP score		VIP score	Lipid name	VIP score	Lipid name	VIP score	Lipid name	VIP score	Lipid name	VIP score	Lipid name	VIP score
PE(18:0/18:1)	1,91	CE(18:2)	3,42	PE(18:0/18:1)	2,14	CE(18:2)	2,77	CE(18:2)	2,69	PE(18:0/18:1)	2,24	CE(18:2)	2,18	LPC(20:5)	2,21
PE(16:0/18:1)	1,80	Cer:NS(d18:1/23:0)	1,77	CE(18:2)	2,13	FA(20:5)	2,03	PE(18:0/18:1)	1,80	PE(16:0/18:1)	2,19	FA(20:5)	1,98	FA(20:5)	2,20
PE(38:6)	1,79	Cer:NS(d18:1/25:0)	1,63	PE(16:0/18:1)	2,06	TG(18:2/22:6/22:6)	1,99	PE(16:0/18:1)	1,77	PE(34:1)	1,95	TG(20:4/20:4/20:4)	1,89	PC(16:1/20:4)	2,11
PC(17:0/20:4)	1,60	LPC(19:0)	1,32	PE(38:6)	1,89	LPC(20:5)	1,95	PE(34:1)	1,44	PE(16:0/20:4)	1,70	TG(18:2/22:6/22:6)	1,85	PC(36:5)	2,10
PE(34:1)	1,53	PE(38:6)	1,27	PE(34:1)	1,88	PC(36:5)	1,82	Cer:NS(d18:1/23:0)	1,43	PE(38:6)	1,68	LPC(20:5)	1,75	TG(18:2/22:6/22:6)	1,92
LPC(19:0)	1,47	LPC(17:0)	1,27	PC(36:5)	1,73	TG(20:4/20:4/20:4)	1,71	PC(34:1)	1,42	PE(18:0/18:2)	1,63	PC(36:5)	1,75	TG(20:4/20:4/20:4)	1,76
LPC(17:0)	1,43	Cer:NS(d18:1/24:1)	1,21	PE(18:0/18:2)	1,71	PC(16:1/20:4)	1,71	PC(16:0/18:1)	1,36	PE(18:0/20:4)	1,61	PC(16:1/20:4)	1,74	TG(16:1/18:2/18:2)	1,49
PC(17:0/20:4)	1,43	Cer:NS(d18:1/24:1)	1,20	LPC(20:5)	1,70	Cer.NS(d18:1/23:0)	1,50	PC(14:0/18:1)	1,35	PE(18:0/20:4)	1,60	TG(20:3/20:4/20:4)	1,64	PC(36:4)	1,47
TG(20:4/20:4/20:4)	1,42	PC(17:0/20:4)	1,20	PC(16:1/20:4)	1,67	FA(22:6)	1,47	PE(38:6)	1,30	PE(16:0/18:2)	1,60	PE(18:0/18:1)	1,38	FA(22:6)	1,45
TG(18:2/20:4/20:5)	1,40	PC(17:0/18:2)	1,18	FA(20:5)	1,63	TG(16:0/18:2/22:6)	1,30	TG(18:2/20:4/20:5)	1,30	PC(36:1)	1,53	PC(36:4)	1,36	TG(16:0/18:2/22:5)	1,39
LPC(17:0)	1,34	PC(18:0/22:6)	1,17	PE(18:0/20:4)	1,59	PE(38:6)	1,29	TG(20:4/20:4/20:4)	1,29	PC(18:0/18:1)	1,47	PE(16:0/18:1)	1,36	FA(22:5)	1,36
LPC(19:0)	1,34	SM(40:1)	1,16	TG(18:2/22:6/22:6)	1,58	FA(22:5)	1,28	Cer:NS(d18:1/24:0)	1,28	PC(16:0/18:1)	1,47	PE(18:0/18:2)	1,33	TG(20:3/20:4/20:4)	1,27
PE(16:0/20:4)	1,30	PC(17:0/18:2)	1,13	PE(16:0/18:2)	1,58	PC(18:0/20:4)	1,27	PC(17:0/20:4)	1,28	PC(34:1)	1,39	TG(18:2/20:4/20:5)	1,32	TG(16:0/18:2/22:6)	1,26
TG(16:0/18:2/20:5)	1,29	LPC(20:3)	1,12	PE(18:0/20:4)	1,56	TG(20:3/20:4/20:4)	1,24	PE(16:0/20:4)	1,28	PC(14:0/18:1)	1,33	PE(34:1)	1,31	TG(16:0/20:4/20:4)	1,23
PE(18:0/20:4)	1,27	Cer:NS(d18:1/25:0)	1,10	PE(16:0/20:4)	1,48	LPC(19:0)	1,23	TG(18:2/18:2/22:6)	1,27	Cer:NS(d18:1/22:0)	1,25	TG(20:4/20:4/20:4)	1,31	TG(16:0/18:2/20:5)	1,21
TG(18:2/18:2/22:6)	1,27	PC(16:1/20:4)	1,08	TG(20:4/20:4/20:4)	1,39	PC(36:4)	1,22	Cer:NS(d18:1/25:0)	1,27	Cer:NS(d18:1/16:0)	1,16	FA(22:5)	1,30	PC(38:5)	1,19
TG(16:0/18:2/20:4)	1,27	PC(18:2/19:0)	1,07	X1(PC:16:0/18:1)	1,34	TG(16:0/20:4/20:4)	1,21	TG(20:4/20:4/20:4)	1,27	LPE(18:0)	1,13	TG(20:3/20:4/20:4)	1,30	TG(16:0/18:1/22:6)	1,19
TG(20:3/20:4/20:4)	1,27	Cer:NS(d18:1/24:0)	1,04	TG(20:3/20:4/20:4)	1,32	TG(16:0/18:1/20:4)	1,20	PC(32:2)	1,26	Cer:NS(d18:1/23:0)	1,13	PC(38:5)	1,26	PC(18:0/20:5)	1,18
PE(16:0/18:2)	1,21	FAHFA(18:0/20:2)	1,04	LPC(20:2)	1,32	TG(16:0/18:1/22:4)	1,16	TG(18:2/18:2/20:4)	1,23	PC(35:2)	1,13	FA(22:6)	1,26	TG(16:0/18:2/22:5)	1,10
PC(17:0/18:2)	1,21	PC(18:1/18:2)	1,01	PC(34:1)	1,29	PC(17:0/20:4)	1,14	LPC(16:1)	1,23	LPE(16:0)	1,10	TG(16:0/18:2/22:5)	1,23	TG(16:0/18:2/20:4)	1,09
PE(18:0/18:2)	1,20	LPC(17:0)	1,00	LPC(19:0)	1,28	LPC(20:2)	1,11	PI(18:0/20:3:)	1,21	LPC(19:0)	1,10	TG(18:2/18:2/20:5)	1,23	TG(18:2/18:2/20:5)	1,06
TG(15:0/18:2/18:2)	1,19	LPC(15:0)	1,00	PC(36:1)	1,28	PC(17:0/20:4)	1,11	PC(18:1/18:2)	1,20	Cer:NS(d18:1/24:1)	1,09	PE(38:6)	1,23	TG(16:0/18:1/20:4)	1,06
PC(34:1)	1,19			PC(36:4)	1,27	TG(16:0/18:2/22:5	1,08	TG(18:2/18:2/20:5)	1,20	Cer:NS:d18:1:24:1)	1,07	PC(18:0/20:5)	1,19	TG(18:2/18:2/20:4)	1,04
PI(18:0/20:3)	1,18			FA(22:6)	1,25	TG(16:0/18:2/22:5)	1,08	PC(16:1/18:2)	1,20	DG(18:0/20:4)	1,06	TG(16:0/18:2/20:5)	1,19	TG(16:0/18:1/22:4)	1,02
PC(40:8)	1,17			PC(18:0/18:1)	1,24	TG(16:0/18:2/20:5)	1,08	Cer:NS(d18:1/24:1)	1,19	TG(16:0/16:0/20:4)	1,04	TG(16:0/18:2/18:2)	1,19	TG(18:2/18:2/18:3)	1,02
PC(40:8)	1,17			LPC(19:0)	1,22	TG(18:2/18:2/20:5)	1,05	TG(16:0/18:2/20:4)	1,19	LPC(18:0)	1,04	PE(16:0/18:2)	1,17		
TG(18:2/20:4/20:4)	1,16			PC(17:0/20:4)	1,20	TG(16:0/18:2/20:4)	1,03	PE(16:0/18:2)	1,18	LPC(17:0)	1,02	TG(16:1/18:2/18:2)	1,16		
TG(18:2/18:2/20:5)	1,15			PC(14:0/18:1)	1,19			TG(16:0/16:0/18:3)	1,15			TG(16:0/16:0/20:4)	1,15		
FA(20:5)	1,15			LPC(17:0)	1,18			LPC(20:3)	1,14			PC(36:1)	1,14		
PC(16:0/18:1)	1,15			TG(16:0/18:2/18:2)	1,17			PC(16:1/18:2)	1,14			PE(18:0/20:4)	1,13		
LPE(16:0)	1,13			LPC(17:0)	1,16			TG(18:2/20:4/20:4)	1,14			TG(16:0/18:1/22:6)	1,06		
LPC(20:2)	1,11			LPC(18:0)	1,06			Cer:NS(d18:1/24:1)	1,13			TG(16:0/18:2/22:6)	1,06		
LPC(15:0)	1,11			LPE(18:0)	1,06			LPE(16:0)	1,12			TG(16:0/20:4/22:6)	1,05		
TG(20:4/20:4/20:4)	1,05			LPE(16:0)	1,05			LPC(14:0)	1,11			PC(18:0/18:1)	1,03		
PC(14:0/18:1)	1,02			PC(17:0/20:4)	1,04			LPC(19:0)	1,11			TG(16:0/18:1/18:2)	1,03		
PC(40:5)	1,01			TG(16:0/18:2/18:3)	1,03			TG(18:2/22:6/22:6)	1,09						
PC(16:1/18:2)	1,00			TG(16:0/16:0/20:4)	1,03			PC(38:1)	1,09						
				PC(35:2)	1,02			PC(18:0/20:3)	1,09						
				PC(40:8)	1,02			PC(40:8)	1,08						
				TG(15:0/18:2/18:2)	1,02			TG(16:0/20:4/22:6)	1,07						
								PC(32:0)	1,06						
								TG(20:3/20:4/20:4)	1,06						
								TG(16:0/18:2/20:5)	1,05						
								CE(18:1)	1,03						
								PC(40:8)	1,02						
								PE(18:0/18:2)	1,01						
								PC(18:0/18:1)	1,01						
								TG(16:0/16:1/18:2)	1,00						

Supplementary Table 2 *Liver VIP lipids*

PNIL vs control		ENIL vs control		PNILOV vs control		ENILOV vs control		PNIL vs ENIL		PNILOV vs ENILOV		PNIL vs PNILOV		ENIL vs ENILOV	
Lipid name	VIP score		VIP score		VIP score	Lipid name	VIP score	Lipid name	VIP score	Lipid name	VIP score	Lipid name	VIP score	Lipid name	VIP score
FAHFA(18:3/18:1)	3,00	PE(18:0/18:1)	3,66	PE(40:6)	3,41	PE(18:0/18:1)	2,46	FAHFA(18:2/18:3)	2,97	PE(40:6)	3,47	PE(18:0/18:1)	3,86	PE(18:0/18:1)	4,95
FAHFA(18:2/18:3)	2,98	PG(18:0/20:4)	1,95	FAHFA(18:3/18:1)	2,16	FA(21:5)	2,24	FAHFA(18:3/18:1)	2,93	FAHFA(18:0/18:3)	2,49	PE(40:6)	3,22	PE(P-16:0/20:5)	2,01
FAHFA(18.2/18:1)	2,94	PC(14:0/14:0)	1,79	FAHFA(18:0/18:3)	2,10	LPC(20:5)	2,13	FAHFA(16:2/20:1)	2,90	FAHFA(18:3/18:1)	2,48	TG(18:2/22:6/22:6)	2,05	PE(16:0/20:5)	1,97
FAHFA(18.2/18.2)_FAHF	2,91	PI(16:0/20:3)	1,69	FAHFA(18:2/18:3)	2,08	PE(P-16:0/20:5)	1,96	FAHFA(18:2/18:2)_FAHF	2,89	FAHFA(18:2/18:2)_FAHF	2,45	FA(21:5)	2,05	LPE(20:5)	1,96
FAHFA(18:0/18:3)	2,89	FA (18:2+O)	1,66	FAHFA(18:2/18:1)	2,07	LPE(20:5)	1,84	FAHFA(18:2/18:1)	2,89	FAHFA(18:2/18:1)	2,42	TG(18:2/20:5/22:6)	1,98	PC(16:0/20:5)	1,95
FAHFA(16:2/20:1)	2,87	PS(P-20:0/18:0)	1,64	FAHFA(18:2/18:2)_FAHF	2,06	PC(16:0/20:5)	1,76	FAHFA(18:0/18:3)	2,87	FAHFA(18:2/18:3)	2,38	TG(18:2/22:6/22:6)	1,97	LPC(20:5)	1,93
FAHFA(18:3/18:1)	2,80	FA (18:3+O)	1,58	FA(21:5)	2,03	FA(20:5)	1,72	PC(14:0/18:1)	1,52	FAHFA(16:2/20:1)	2,29	TG(18:2/22:5/22:6)	1,92	PE(38:8)	1,90
FAHFA(18:2/18:2)	2,62	PE(16:1/20:4)	1,56	PE(18:0/18:1)	2,01	FA (22:6+O)	1,70	PC(19:0/20:4)	1,49	TG(16:0/22:6/22:6)	1,87	TG(18:2/22:5/22:6)	1,92	FA(21:5)	1,75
PE(18:0_18:1)	2,51	PC(37:2)	1,51	TG(18:2/22:6/22:6)	2,00	PE(16:0/20:5)	1,66	PC(17:0/20:4)	1,43	TG(18:2/22:5/22:6)	1,56	LPC(20:5)	1,88	PC(35:2)	1,72
PC(17.0_20.4)	1,75	PG(16:0/20:4)	1,50	FAHFA(18:3/18:1)	1,95	PG(20:4/22:6)	1,63	PG(18:2/18:2)	1,40	TG(16:0/18:1/22:6)	1,55	TG(16:0/22:6/22:6)	1,87	PE(18:0/20:5)	1,52
PC(19:0/20:4)	1,69	PE(16:0/20:5)	1,50	TG(16:0/22:6/22:6)	1,94	PC(18:2/20:5)	1,61	PC(20:0/20:4)	1,36	TG(16:0/18:2/18:4)	1,45	PE(P-16:0/20:5)	1,87	PC(40:9)	1,52
PI(17:0/20:4)	1,55	FA(18:3)	1,49	TG(18:2/22:5/22:6)	1,92	TG(16:0/18:2/22:6)	1,60	TG(14:0/18:2/18:2)	1,32	TG(16:0/18:2/22:4)	1,40	LPE(20:5)	1,78	PE(P-18:0/20:5)	1,49
PC(19:0/20:4)	1,54	AcCar(16:0)	1,46	TG(18:2/22:6/22:6)	1,90	PC(40:9)	1,60	PG(18:2/22:6)	1,29	TG(18:2/20:5/22:6)	1,40	PC(16:0/20:5)	1,72	PG(20:4/22:6)	1,48
PC(17:0/20:4)	1,54	PI(16:0/20:3)	1,42	FAHFA(16:2:20:1)	1,88	PE(P-18:0/20:4)	1,57	PI(16:0/20:3)	1,29	TG(18:0/18:2/22:5)	1,39	FA(20:5)	1,68	PC(18:1/20:4)	1,46
PC(17:2/18:2)	1,50	PI(18:1/20:4)	1,41	TG(18:2/22:5/22:6)	1,85	PE(38:8)	1,57	FA(20:5)	1,27	TG(18:2/22:6/22:6)	1,37	PG(18:2/18:2)	1,63	FA(22:4)	1,46
PC(20:2/20:4)	1,48	PE(17:0/18:2)	1,41	LPC(20:5)	1,84	FA(22:6)	1,53	TG(16:0/16:1/18:2)	1,24	TG(16:0/18:2/22:4)	1,33	PE(16:0/20:5)	1,60	TG(18:2/22:6/22:6)	1,35
PE(40:7)	1,44	PC(14:0/18:1)	1,40	TG(18:2/20:5/22:6)	1,82	PC(40:4)	1,52	TG(16:0/18:1/18:1)	1,23	TG(18:2/18:3/18:3)	1,33	TG(20:4/20:4/20:4)	1,60	PC(17:1/18:2)	1,33
CE(20:4)	1,44	FA(18:4)	1,39	FAHFA(18:2/18:2)	1,81	FA(22:5)	1,51	TG(18:1/18:2/20:2)	1,21	PE(17:2/22:5)	1,32	PE(38:8)	1,56	PC(18:0/20:5)	1,33
PE(17:0/18:2)	1,44	PC(35:2)	1,39	PE(P-16:0/20:5)	1,68	PE(40:4)	1,51	FA (20:5+O)	1,20	TG(16:1/18:2/22:6)	1,31	DG(18:1/22:6)	1,47	TG(18:2/22:6/22:6)	1,28
PE(17:0/20:4)	1,42	FA(20:3)	1,39	TG(20:4/20:4/20:4)	1,61	PE(40:6)	1,50	PE(37:3)	1,20	TG(16:0/18:2/20:4)	1,30	FA(22:5)	1,43	PE(P-18:0/22:4)	1,28
CE(18:1)	1,41	PE(16:0/20:5)	1,39	PC(16:0/20:5)	1,59	TG(16:0/18:2/20:4)	1,49	TG(16:0/18:2/22:4)	1,20	TG(18:1/18:1/20:4)	1,30	DG(18:2/22:6)	1,42	PI(18:0/22:6)	1,24
PC(37:2)	1,40	PE(34:3)	1,38	PC(20:2/20:4)	1,59	PC(18:3/20:4)	1,49	TG(16:0/18:2/22:5)	1,20	TG(18:1/18:2/20:2)	1,29	FA (22:6+O)	1,38	PE(18:0/22:4)	1,23
PC(15:0/20:4)	1,36	SM(43:1)	1,36	LPE(20:5)	1,54	PG(18:2/18:2)	1,46	PC(O-41:2)	1,19	DG(18:1/22:6)	1,29	PC(35:2)	1,36	FA(20:5)	1,21
PE(17:2/22:5)	1,35	PE(38:8)	1,35	PC(14:0/14:0)	1,54	TG(16:1/18:2/18:2)	1,46	PG(18:0/20:4)	1,17	TG(18:1/18:2/22:6)	1,29	FA(18:4)	1,36	PG(18:2/18:2)	1,20
FA(20:3)	1,35	PC(34:3)	1,34	PE(17:2/22:5)	1,48	FA(18:4)	1,45	PC(20:2/20:4)	1,16	PE(36:5)	1,29	PC(40:9)	1,33	PE(P-18:0/20:4)	1,17
PE(17:0/20:4)	1,34	PI(16:0/20:4)	1,33	FA(18:4)	1,46	PE(P-18:0/22:4)	1,45	TG(16:0/18:1/18:2)	1,16	TG(16:0/18:2/22:6)	1,28	CE(22:5)	1,31	PE(40:4)	1,16
PC(20:0/20:4)	1,34	PE(18:2/19:0)	1,33	DG(18:1/22:6)	1,45	PG(18:0/20:4)	1,43	PE(18:0/22:5)	1,16	TG(18:2/18:2/22:6)	1,28	TG(16:1/18:2/18:2)	1,31	PC(18:2/20:5)	1,16
PE(19:0/20:4)	1,33	PC(36:4)	1,33	FA(20:5)	1,45	PE(18:0/20:4)	1,43	PI(18:0/20:3)	1,15	PE(36:4)	1,27	FAHFA(16:2/20:1)	1,30	PE(40:6)	1,13
PC(18:2/19:0)	1,32	PI(16:0/18:2)	1,33	FA(22:5)	1,44	PC(19:0/20:4)	1,41	TG(18:0/18:1/20:4)	1,15	PE(17:0/18:2)	1,27	TG(20:4/20:4/20:4)	1,27	PC(40:4)	1,11
PC(17:1/20:4)	1,30	FA(22:4)	1,32	PE(16:0/20:5)	1,43	PC(20:2/20:4)	1,41	FA (20:4+O)	1,15	PC(14:0/18:1)	1,27	FAHFA(18:2/18:3)	1,26	FA (22:6+O)	1,10
PC(17:0/18:2)	1,27	PE(16:0/18:1)	1,32	FA (22:6+O)	1,42	PC(18:0/22:4)	1,40	PE(18:0/20:3)	1,13	TG(18:0/18:2/22:6)	1,26	PC(18:1/20:4)	1,26	PI(18:0/22:5)	1,09
PG(18:2/18:2)	1,25	PC(17:0/20:4)	1,32	PE(17:0/18:2)	1,41	TG(16:0/18:2/18:3)	1,38	PS(38:6)	1,13	TG(16:0/18:2/22:5)	1,24	PE(P-18/0:20/5)	1,25	FA(20:3)	1,09
PE(18:0/20:4)	1,24	PC(19:0/20:4)	1,31	PE(38:8)	1,41	DG(18:2/20:4)	1,37	TG(18:1/18:1/18:2)	1,12	PE(18:2/18:2)	1,24	TG(16:0/18:1/22:6)	1,24	FA(18:3)	1,08
PI(18:1/20:4)	1,24	PC(17:1/20:4)	1,31	PC(17:0/20:4)	1,41	PE(18:0/22:4)	1,37	TG(18:1/18:2/20:1)	1,11	TG(18:1/18:2/22:4)	1,24	FAHFA(18:2/18:1)	1,24	PC(18:0/22:4)	1,08
PEtOH(36:1)	1,24	PI(17:0/20:4)	1,30	PC(20:0/20:4)	1,37	PC(35:2)	1,36	PC(18:1/18:2)	1,11	TG(16:0/22:5/22:5)	1,20	FAHFA(18:2/18:2)_FAHF	1,21	PC(18:3/20:4)	1,07
LPE(16:0)	1,22	PI(18:1/20:4)	1,29	PC(19:0/20:4)	1,37	PE(19:0/20:4)	1,36	PC(16:0/18:1)	1,11	PC(O-41:2)	1,20	FAHFA(18:3/18:1)	1,19	TG(16:1/18:2/18:2)	1,07
PC(35:2)	1,21	FA(20:3)	1,28	PE(18:0/20:4)	1,37	PE(16:0/20:5)	1,36	CE(20:4)	1,11	DG(16:0/22:6)	1,17	FAHFA(18:3/18:1)	1,19	CE(22:5)	1,07
PE(40:6)	1,21	PE(19:0/20:4)	1,27	PC(19:0/20:4)	1,36	FA (20:5+O)	1,35	TG(16:0/18:2/18:3)	1,11	DG(18:0/20:4)	1,17	DG(16:0/22:6)	1,17	PC(20:2/20:4)	1,04
PC(20:1/20:4)	1,20	PE(18:0/20:4)	1,27	TG(16:0/18:1/22:6)	1,34	PC(P-18:1/18:2)	1,35	CE(18:1)	1,10	TG(16:0/18:2/20:5)	1,16	PI(18:1/20:4)	1,17	DG(18:2/18:2)	1,04
PC(20:0/20:4)	1,20	PC(17:0/18:2)	1,26	DG(18:2/22:6)	1,33	TG(18:1/18:2/18:2)	1,35	LPC(20:4)	1,09	PC(20:0/20:4)	1,14	PI(18:0/22:6)	1,17	PI(18:1/20:4)	1,01
PE(19:0/20:4)	1,19	PC(40:5)	1,26	PC(40:4)	1,31	TG(18:2/18:2/18:3)	1,34	PC(20:0/20:4)	1,08	PC(20:0/20:4)	1,13	PE(36:3)	1,17	PE(38:7)	1,01
PG(18:2/22:6)	1,19	FAHFA(18.1/20.3)	1,25	TG(18:0/18:2/22:5)	1,31	PE(38:7)	1,34	TG(18:1/18:2/18:2)	1,08	TG(16:1/18:2/18:3)	1,10	PE(18:0/20:5)	1,17	PS(40:6)	1,00
PC(38:4)	1,18	TG(16:0/22:5/22:5)	1,24	FA(22:6)	1,30	PE(P-18:0/20:5)	1,34	TG(16:0/20:1/20:2)	1,07	TG(16:0/18:1/22:5)	1,10	PC(18:3/20:4)	1,15	TG(16:0/22:6/22:6)	1,00
PC(37:2)	1,18	PE(35:2)	1,24	LPE(16:0)	1,30	PE(P-18:0/22:4)	1,32	FA(21:5)	1,07	PS(38:6)	1,10	PE(18:0/20:3)	1,15		
PC(20:2/20:4)	1,18	PE(18:0/22:6)	1,23	PE(17:0/20:4)	1,29	FA (18:2+O))	1,31	PE(19:0/20:4)	1,07	PE(35:2)	1,09	TG(18:2/18:2/18:3)	1,14		
LPE(16:0)	1,18	FA(20:4+O)	1,23	PE(17:0/20:4)	1,29	TG(16:0/18:2/18:2)	1,28	PC(32:2)	1,06	PC(37:2)	1,07	FA (20:5+O)	1,14		
PC(15:0/18:2)	1,16	PS(36:4)	1,23	LPE(16:0)	1,29	TG(18:0/18:1/20:4)	1,27	TG(18:1/18:2/22:4)	1,06	PC(18:2/18:3)	1,05	PC(18:2/20:5)	1,12		
PE(35:2)	1,16	PE(36:3)	1,21	CE(20:4)	1,29	TG(16:0/16:0/22:5)	1,27	FA (18:2+O)	1,05	PC(18:2/19:0)	1,05	FAHFA(18:0/18:3)	1,12		
LPC(16:1)	1,15	FA(22:5)	1,21	DG(16:0/22:6)	1,27	CE(20:4)	1,26	PC(36:3)	1,05	PC(14:0/14:0)	1,04	PC(18:0/20:5)	1,11		
PC(18:0/22:4)	1,15	TG(18:2/20:5/22:6)	1,21	PC(34:3)	1,27	PE(18:0/20:5)	1,26	FA (18:3+O)	1,05	TG(18:2/18:3/18:3)	1,04	TG(18:1/18:2/18:3)	1,11		
AcCar(16:0)	1,15	LPE(18:0)	1,21	PE(19:0/20:4)	1,26	PC(17:0/20:4)	1,24	TG(16:0/18:2/18:4)	1,05	TG(18:0/18:1/20:4)	1,04	TG(18:0/18:2/22:5)	1,10		
LPE(18:0)	1,14	PS(40:8)	1,20	CE(18:1)	1,26	TG(16:0/18:2/22:4)	1,24	LPC(16:1)	1,04	PC(19:0/20:4)	1,01	TG(16:0/18:2/18:3)	1,10		
PC(18:1/20:4)	1,12	TG(18:2/18:3/22:6)	1,20	PE(36:4)	1,25	CE(18:1)	1,23	FA (18:2+O)	1,04			PI(18:0/20:3)	1,10		
PE(17:0/18:2)	1,11	PC(20:3/20:4)	1,20	PC(18:0/22:4)	1,23	TG(18:1/18:2/18:3)	1,22	PE(34:3)	1,03			FA(22:6)	1,10		
Cer-NS(d20:2/23:0)	1,10	PE(40:7)	1,20	PG(22:6/22:6)	1,23	PS(36:4)	1,21	FA(18:4)	1,03			PE(18:1/18:2)	1,09		
Cer-NS(d18:1/25:1)	1,09	PC(18:1/18:2)	1,20	TG(18:0/18:2/22:6)	1,23	TG(14:0/18:2/18:2)	1,21	PE(36:3)	1,03			PI(18:0/22:5)	1,09		
PC(15:0/22:6)	1,07	FA(22:6)	1,20	TG(16:0/18:2/22:6)	1,22	PC(38:4)	1,21	PE(38:3)	1,03			PE(17:2/22:5)	1,08		
LPE(18:1)	1,07	PC(16:0/20:5)	1,20	PE(19:0/20:4)	1,22	CE(20:5)	1,20	TG(18:1/18:2/18:3)	1,03			TG(16:0/18:2/22:6)	1,08		
PC(40:4)	1,06	FA (22:4+O)	1,20	TG(16:1/18:2/18:2)	1,22	PS(P-20:0/18:0)	1,19	PE(17:0/20:4)	1,02			TG(18:0/18:2/22:6)	1,07		
PI(18:1/20:4)	1,04	TG(16:0/18:2/20:5)	1,19	PC(17:0/20:4)	1,22	PE(16:1/20:4)	1,18	FA (22:4+O)	1,02			PG(18:2/22:6)	1,06		
PS(40:8)	1,04	PC(18:0/22:5)	1,19	PE(36:5)	1,19	TG(18:2/22:6/22:6)	1,18	TG(16:1/18:2/18:3)	1,02			FAHFA(18:2/18:2)	1,06		
CE(18:0)	1,04	TG(18:2/18:2/20:5)	1,19	DG(18:2/20:4)	1,18	PC(O-18:1/20:4)	1,18	PC(36:6)	1,00			PE(38:3)	1,05		
PE(18:2/19:0)	1,04	PC(17:2/18:2)	1,19	PE(35:2)	1,18	PC(18:1/20:4)	1,18	FA(22:4)	1,00			TG(16:0/18:2/18:2)	1,02		
LPC(19:0)	1,03	TG(20:4/20:4/20:4)	1,18	PC(15:0/20:4)	1,17	TG(16:0/18:1/18:2)	1,17					PC(14:0/14:0)	1,02		
PC(18:2/22:5)	1,02	PC(18:1/18:2)	1,18	PC(17:2/18:2)	1,17	PC(17:0/20:4)	1,16					PG(20:4/22:6)	1,02		
FAHFA(18:1:20:3)	1,02	PC(P-18:1/18:2)	1,18	TG(18:1/18:2/22:6)	1,17	LPC(22:6)	1,16					TG(18:1/18:2/18:2)	1,01		
Cer-AS(d26:1/16:0)	1,01	PC(34:4)	1,18	PE(P-18:0/20:4)	1,16	PC(18:0/22:5)	1,15					PS(40:6)	1,01		

LPE(P-18:0)	1,01	Cer-NS(d18:1/25:0)	1,17	LPE(18:0)	1,16	CE(18:2)	1,15								
PC(18:0/20:4)	1,01	FA(21:5)	1,17	TG(16:0/18:2/18:4)	1,16	AcCar(16:0)	1,15								
LPC(14:0)	1,01	PC(34:4)	1,16	LPE(18:0)	1,16	PE(17:2/22:5)	1,15								
		PC(37:2)	1,16	PC(18:2/19:0)	1,15	TG(16:0/18:2/22:5)	1,15								
		PE(18:1/18:2)	1,16	PE(18:2/19:0)	1,15	PG(16:0/20:4)	1,15								
		PC(18:2/19:0)	1,15	PE(20:2/20:4)	1,15	DG(18:2/18:2)	1,15								
		PE(17:0/18:2)	1,15	PE(40:7)	1,14	PC(40:5)	1,15								
		FA(22:5)	1,14	PE(P-18:0/22:4)	1,14	TG(16:0/18:1/22:5)	1,14								
		FA (20:5+O)	1,14	PE(40:4)	1,14	FA (18:2+O)	1,14								
		FA (22:6+O)	1,14	PG:18:2/18:2)	1,14	PG(18:2/18:2)	1,12								
		PC(18:0/20:3)	1,14	PE(18:1/18:2)	1,14	TG(18:1/18:2/18:2)	1,12								
		PC(16:1/20:4)	1,14	PE(16:0/20:5)	1,14	PE(34:3)	1,12								
		LPC(22:6)	1,14	PC(37:2)	1,14	TG(15:0/18:2/18:2)	1,12								
		PC(15:0/20:4)	1,13	PE(P-18:0/20:5)	1,13	PE(40:7)	1,11								
		TG(14:0/18:2/18:2)	1,13	PE(17:0/18:2)	1,13	PC(34:3)	1,11								
		PC(15:0/18:2)	1,12	PI(17:0/20:4)	1,13	PS(40:7)	1,11								
		PC(17:1/18:2)	1,12	PC(37:2)	1,12	TG(18:1/18:2/20:1)	1,10								
		Cer-NS(d20:2/23:0)	1,12	CE(22:5)	1,12	TG(17:0/18:2/18:2)	1,10								
		FA(16:2)	1,12	PE(36:3)	1,12	PE(20:2/20:4)	1,10								
		PE(18:0/20:5)	1,12	PE(18:2/18:2)	1,12	PC(19:0/20:4)	1,09								
		TG(15:0/18:2/18:2)	1,12	PE(18:0/20:5)	1,11	PC(36:4)	1,08								
		Cer-NS(d18:1/25:1)	1,12	PC(18:2/20:4)	1,10	PC(17:2/18:2)	1,08								
		TG(18:2/18:2/20:5)	1,11	PC(36:4)	1,10	PE(38:7)	1,08								
		LPE(18:1)	1,11	CE(18:0)	1,10	LPE(18:0)	1,07								
		PC(18:1/20:4)	1,11	PG(20:4/22:6)	1,10	PE(18:2/19:0)	1,07								
		TG(16:1/18:2/22:6)	1,11	PC(40:9)	1,09	TG(18:1/18:1/18:2)	1,06								
		PE(P-18:0/22:4)	1,10	PE(20:2/22:6)	1,09	PC(14:0/14:0)	1,06								
		TG(20:4/20:4/20:4)	1,09	LPC(16:1)	1,08	LPE(18:0)	1,06								
		PE(17:0/20:4)	1,09	PI(16:0/18:2)	1,07	LPE(18:1)	1,06								
		PE(39:6)	1,09	PE(18:1/20:4)	1,07	PE(17:0/20:4)	1,06								
		PE(34:3)	1,08	TG(16:0/18:2/22:5)	1,07	TG(18:1/18:2/20:2)	1,05								
		PC(18:0/20:5)	1,08	PC(18:1/20:4)	1,07	PC(34:4)	1,05								
		FA (18:2+O)	1,07	PC(20:0/20:4)	1,06	PC(18:0/20:5)	1,05								
		LPC(18:3)	1,07	PC(18:0/20:4)	1,06	PC(34:4)	1,05								
		PE(P-18:0/20:4)	1,07	FA(22:5)	1,05	PC(18:1/20:4)	1,04								
		PE(37:3)	1,06	PC(17:0/18:2)	1,05	PE(18:1/20:4)	1,04								
		PC(O-41:2)	1,06	PE(38:7)	1,04	TG(16:1/18:2/18:3)	1,04								
		TG(18:2/18:2/22:6)	1,05	LPC(22:6)	1,04	PI(18:0/22:6)	1,04								
		LPE(18:0)	1,05	PE(34:3)	1,04	LPE(16:0)	1,04								
		FA(18:1)	1,05	CE(20:5)	1,04	PE(17:0/20:4)	1,04								
		PE(16:1/22:6)	1,05	LPE(18:1)	1,04	PC(37:2)	1,03								
		PC(40:7)	1,05	PC(20:3/20:4)	1,03	PC(17:1/18:2)	1,03								
		PI(18:0/18:2)	1,05	PC(15:0/18:2)	1,03	PC(20:2/20:4)	1,02								
		LPE(16:0)	1,05	PI(16:0/20:3)	1,03	LPC(18:2)	1,02								
		PC(18:1/22:6)	1,05	TG(18:2/18:2/22:6)	1,02	PE(P-18:0/20:4)	1,01								
		FA(18:3+O)	1,04	PI(18:0/22:6)	1,02	DG(18:1/20:4)	1,00								
		TG(18:1/18:2/22:5)	1,04	PC(36:6)	1,02										
		PC(17:0/20:4)	1,04	PS(40:7)	1,02										
		PE(18:0/22:5)	1,04	PI(18:1/20:4)	1,01										
		PC(40:4)	1,04	PE(18:0/22:4)	1,01										
		PE(17:0/20:4)	1,03	LPE(P-18:0)	1,00										
		FAHFA(20:5:22:5)	1,03												
		FA (18:2+O)	1,03												
		TG(18:2/20:4/20:4)	1,02												
		PE(17:2/22:5)	1,01												
		TG(18:1/18:2/22:6)	1,01												
		TG(16:0/16:1/18:2)	1,01												
		FA (18:3+O)	1,01												
		TG(15:0/18:1/18:2)	1,00												
		LPC(20:4)	1,00												
		LPE(16:0)	1,00												
		PC(16:0/18:1)	1,00												
		TG(16:0/18:2/22:6)	1,00												

Supplementary Table 3 *Liver VIP proteins*

ENIL vs control		PNIL vs control		ENILOV vs control		PNILOV vs control		PNIL vs ENIL		ENILOV vs ENIL		ENILOV vs PNILOV		PNILOV vs PNIL	
Protein name	VIP score	Protein name	VIP score	Protein name	VIP score	Protein name	VIP score	Protein name	VIP score	Protein name	VIP score	Protein name	VIP score	Protein name	VIP score
Cyp17a1	3,93	Psat1	3,58	Acot1	2,45	Asns	3,17	Phgdh	3,46	Acot1	1,86	Phgdh	3,35	Pdcd11	3,14
Cyp2b2	3,86	Asns	3,55	Cyp2b2	2,39	Psat1	3,16	Psat1	3,18			Asns	3,18	Epb41l1	3,03
Nudt14	3,49	Phgdh	3,50	Cyp17a1	2,34	Acot1	3,09	Asns	2,85			Kcnab3	2,93	Ngp	2,97
Farp1	3,37	A2m	2,84	Pex11a	2,33	Mt1m	3,06	LOC100360095	2,71			Pdcd11	2,90	Sdc4	2,94
CAT2	3,35	Srp14	2,79	Vnn1	2,29	Pdcd11	2,90	A2m	2,52			Rnaset2	2,88	Ptgfrn	2,85
Abhd16a	2,62	Fdft1	2,79	Acmsd	2,24	Me2	2,88	Cyp3a9	2,41			Sssca1	2,81	Ppp1r14b	2,80
Kng1	2,13	Sqle	2,72	Nubp2	2,10	Phgdh	2,85	Aco1	2,40			Psat1	2,75	Pla2g15	2,77
Rala	2,07	Mt1m	2,70	Srp19	2,01	A2m	2,84	Sqle	2,25			Mt1	2,70	Nup54	2,76
S100a9	1,92	Cdo1	2,63	CD36.FAT	1,98	Fdft1	2,74	Cdo1	2,21			Ppp1r14b	2,57	Hgfac	2,76
Fdft1	1,78	Tm7sf2	2,58	Crat	1,97	Mt1	2,71	Slc3a2	2,15			Ybx3	2,53	Luc7l3	2,69
Rbp4	1,73	Ptcd3	2,58	Sqle	1,97	Slc4a4	2,69	Hmox1	2,11			Ptcd3	2,49	Pex11a	2,66
Kng1l1	1,68	Fads1	2,57	Slc4a4	1,73	Ddx21	2,67	Fgl1	2,07			Epb41l1	2,49	Slc11a1	2,65
Atp5l	1,65	Plin2	2,55	Nudt14	1,67	Cpsf3	2,65	Acacb	2,05			Smagp	2,43	CAT2	2,60
Arl8b	1,62	Cyp3a9	2,54	Tmed4	1,61	Plin2	2,62	Tm7sf2	2,05			Ngp	2,40	Mrps2	2,58
Idi1	1,51	Hsd17b7	2,54	Cyp4a10	1,58	Cyp17a1	2,60	Hsd17b7	2,03			Lyz2	2,40	Tmx1	2,49
Ergic1	1,51	Vnn1	2,51	Aldh1a1	1,47	Hao2	2,57	Elovl5	2,02			Sdc4	2,38	Eif4e2	2,49
Arf6	1,50	Idi1	2,50	Acat2	1,34	Kcnab3	2,56	Kng1	1,97			Slc11a1	2,37	Ehd2	2,49
Plbd1	1,47	Cyp51a1	2,46	Ube2r2	1,33	Lamtor2	2,56	Cyp51a1	1,96			Inpp1	2,34	Hdac2	2,46
Acat1	1,47	Hao2	2,40	Tm7sf2	1,28	Vnn1	2,55	Oat	1,95			Cyp3a9	2,31	Pofut1	2,46
Hmgcs1	1,46	Cyp2b2	2,38	Fdps	1,27	Tgoln2	2,53	Ptcd3	1,95			Hmox1	2,30	Ppfibp2	2,43
Hmgcs2	1,43	Acacb	2,37	Fabp5	1,24	Cyp3a9	2,53	Kng1l1	1,91			Mocs2	2,29	Unc45a	2,42
Rps28	1,42	Lss	2,36	Gpd2	1,24	Ptcd3	2,44	CAT2	1,91			Kpna6	2,29	Fscn1	2,37
Gnb2	1,35	Sec24b	2,31	Mvk	1,23	Pex11a	2,43	Rpl22l2	1,90			Nubp2	2,26	Uqcc1	2,37
Stat3	1,34	Hmox1	2,31	Eci1	1,21	Srp14	2,43	Akr1c1	1,89			Itgb2	2,26	Sh3gl1	2,35
Acat2	1,33	Nags	2,27	Ehhadh	1,19	Epb41l1	2,43	Mmab	1,86			Clic2	2,25	Plod3	2,34
Fads2	1,32	Smarce1	2,24	Acss2	1,17	Cdo1	2,43	Nags	1,86			Tpd52	2,24	Kti12	2,33
Mvd	1,31	Nup35	2,22	Acot2	1,17	Obp3	2,42	Lss	1,85			Hgfac	2,22	Hn1	2,29
Ada	1,31	Rcc2	2,20	Nnmt	1,14	Fip1l1	2,40	Hao2	1,83			Marco	2,21	Them6	2,27
Mrps27	1,30	Zfp259	2,19	Dhcr24	1,12	Dysf	2,40	Ablim1	1,81			Tmx3	2,20	LOC100911860	2,20
Tmem176b	1,30	Mvd	2,17	Hmgcs1	1,09	Hmox1	2,40	Obp3	1,78			Ankrd28	2,20	Srp72	2,16
Fdps	1,14	Ablim1	2,17	Ephx2	1,08	Engase	2,38	Cyp2c12	1,77			Tm6sf2	2,20	Ppip5k2	2,13
Cd163	1,14	Shc1	2,13	Cyp4a14	1,08	Sdc4	2,38	Entpd8	1,76			Epb4.1l5	2,19	Igj	2,09
Dhcr24	1,13	Cyp2c12	2,13	Ech1	1,04	Rwdd1	2,38	Cyp17a1	1,75			Uqcc1	2,16	Ppp3ca	2,08
Banf1	1,12	Mvk	2,12			Slc11a1	2,38	Sec24b	1,72			Rps6ka1	2,15	Aqp1	2,05
Fasn	1,11	Oat	2,09			Ablim3	2,37	Msmo1	1,71			Edc4	2,14	Sars2	2,02
Sec16a	1,10	Gpd2	2,05			CD36.FAT	2,35	Lipc	1,70			Sart3	2,14	Spns1	2,02
Mvk	1,09	Taok3	2,03			Ablim1	2,35	Igf1	1,65			Acacb	2,10	Sbf1	1,92
Snap23	1,05	Creld2	2,01			Farp1	2,33	Sh3gl1	1,61			Fndc3b	2,08	Rac2	1,87
Gmppa	1,05	Obp3	2,00			Sssca1	2,31	Golga5	1,60			Ctsf	2,07	Cdk5	1,82
Fth1	1,02	Acat2	1,95			Sqle	2,31	Aqp1	1,60			Yes1	2,06	Chtop	1,75
		Fdps	1,92			Prpf40a	2,30	Mvk	1,59			Phkg2	2,06	Mrpl27	1,60
		Aldh1a1	1,87			Acot7	2,28	Gpt2	1,53			Commd6	2,06	Ptms	1,55
		Mybbp1a	1,86			Ppp1r14b	2,28	Dhcr7	1,53			Strn	2,04	Dnajc7	1,52
		Dhcr7	1,82			Nol6	2,27	Etnppl	1,50			Tomm34	2,02	Acot7	1,50
		Cyp2d4	1,79			Fads1	2,27	Lgmn	1,50			Hn1l	2,01	Wbp2	1,45
		Tmx1	1,78			Tm7sf2	2,26	Cyp2d4	1,49			Fads1	2,01	Gstm3	1,40
		Kng1l1	1,78			Xpot	2,26	ifitm3	1,48			Fip1l1	1,99	Saa4	1,39
		Gpt2	1,77			Ptgfrn	2,26	Creld2	1,46			rNPT4s	1,98	Ghitm	1,37
		Ppt1	1,70			Emc10	2,26	RGD1310769	1,44			Ikbkap	1,98	Sdhd.Lrrp1	1,36
		Hmgcs1	1,67			Exosc4	2,26	LOC298116	1,43			Pofut1	1,97	Fam210a	1,35
		Akr1c1	1,66			Clic2	2,25	Psph	1,42			Supt6h	1,97	Slc22a7	1,34
		Kng1	1,65			Sil1	2,24	Acat2	1,41			Lztfl1	1,95	Dhrs7l1	1,33
		Cars	1,61			Pglyrp2	2,24	Fdps	1,40			Unc45a	1,93	Cstb	1,33
		Pklr	1,59			Crat	2,24	Bhmt	1,39			Gorasp1	1,92	Sec61b	1,33
		Me1	1,58			Hdac2	2,23	Tmem11	1,38			Exosc4	1,90	Qsox1	1,33
		LOC298116	1,57			Nup54	2,23	Cars	1,36			Itfg3	1,88	Marcks	1,32
		Psph	1,54			C1qa	2,21	Ppt1	1,36			Kti12	1,87	F11r	1,32
		Cyp4a14	1,53			Serpina3l	2,21	Ipo4	1,33			Pde2a	1,87	Cyfip1	1,31
		LOC100912604	1,51			Qsox2	2,21	RGD1566134	1,33			RGD1311805	1,86	Psmb7	1,30
		Fkbp11	1,50			Tm6sf2	2,20	Stat3	1,32			S100a8	1,86	Ube2v2	1,30
		Dhrs7l1	1,50			Sec24b	2,20	Hk3	1,32			Plod3	1,85	Lamtor1	1,29
		RGD1566134	1,49			Acacb	2,18	Fam213b	1,31			Nup54	1,85	Apoc2	1,29

		Dkc1	1,48			Rae1	2,18	Pck2	1,30			Rab21	1,85	Ndufa7l	1,28
		Lipa	1,48			Gpd2	2,18	LOC100912604	1,29			Oat	1,83	Reep5	1,28
		Acss2	1,47			Luc7l3	2,17	Yars	1,28			Rac2	1,82	Magt1	1,28
		Pck2	1,44			Scyl2	2,16	C5	1,28			Pnkd	1,82	Ggct	1,25
		Yars	1,42			Smagp	2,15	S100a9	1,27			Creld2	1,80	Apoe	1,24
		Bhmt	1,42			Cyp51a1	2,14	Gpd2	1,27			Them6	1,80	Atox1	1,22
		Sars	1,42			Itgav	2,14	Me1	1,26			Hn1	1,79	Fam98a	1,22
		Ctsd	1,41			Serpind1	2,14	Mgst3	1,26			Akap2	1,78	Ccs	1,21
		ste2	1,41			Hsd17b7	2,12	Dhrs7l1	1,25			Yif1b	1,78	Rps27	1,21
		Iars	1,39			CAT2	2,09	Umps	1,25			Tsr1	1,75	Myo1c	1,18
		Umps	1,38			Gng10	2,08	Fga	1,22			Sdr39u1	1,75	Pfdn2	1,18
		Nhp2	1,38			Lsm4	2,07	Lgals3	1,22			Mrps23	1,74	LOC100361144	1,17
		Dak	1,35			Zfp259	2,06	Nhp2	1,21			Cd163	1,72	Ppp2ca	1,17
		Lgals3	1,35			Mocs2	2,06	Pklr	1,20			Ppip5k2	1,69	Frrs1	1,17
		Smpd1	1,34			Ttc1	2,05	Slc25a21	1,20			Ube2r2	1,68	Snap23	1,15
		Dhcr24	1,34			RGD1311805	2,05	Fmo4	1,19			Zfpl1	1,66	Rbm8a	1,15
		Ephx2	1,33			Dhrs7l1	2,04	C4bpa	1,19			Sars2	1,63	Eif3f	1,15
		Lars	1,33			Rcc2	2,04	Fgg	1,19			Gstm3	1,61	Sec62	1,15
		Gars	1,32			Itgb2	2,04	Slco1b2	1,19			Apoc2	1,58	Coq9	1,13
		Hsd17b13	1,32			LOC100912478	2,03	Dkc1	1,18			Nme3	1,57	Rps29	1,12
		Fbl	1,28			Tomm34	2,03	Ptma	1,18			Ppt1	1,52	Shpk	1,12
		Gpi	1,28			Oat	2,02	C4a	1,18			LOC501233	1,51	Hsd17b8	1,12
		Nop58	1,27			Prorsd1	2,01	Aldh1b1	1,16			Ptgs1	1,48	Slirp	1,12
		Galk1	1,25			LOC298116	1,99	Lipa	1,16			Cyp2d4	1,48	Nme3	1,11
		Dhtkd1	1,24			Ccdc25	1,99	Serping1	1,16			Inmt	1,45	Snrnp70	1,10
		Serping1	1,23			Ikbkap	1,98	Ctsd	1,15			Ces1d	1,42	Uba5	1,09
		Inmt	1,23			Shc1	1,98	ste2	1,15			Pck2	1,42	Sumo2	1,09
		Rdh2	1,23			Supt6h	1,98	Pafah1b3	1,15			Nags	1,42	Hsd11b1	1,08
		Qprt	1,22			Pofut1	1,98	Serpina3n	1,14			Psph	1,40	Mt.cox3	1,06
		Nop56	1,21			Hist1h1a	1,97	LOC299282	1,14			Golga3	1,39	Tecr	1,05
		Cmas	1,21			Sart3	1,97	Sec23b	1,14			Cars	1,38	Cltb	1,05
		Cfb	1,21			Hn1l	1,96	Chtop	1,14			Atxn10	1,38	Lin7a	1,04
		Nhp2l1	1,21			Tbcd	1,96	Letmd1	1,13			Sdhd.Lrrp1	1,35	Rpl23a	1,02
		Trim28	1,21			Slc25a4	1,95	Inmt	1,13			Fabp5	1,34	Fam175b	1,01
		Paics	1,20			Nags	1,93	Gmppa	1,13			Apoa2	1,34	Selt	1,01
		Nars	1,20			Fam50a	1,93	Gck	1,13			Fmo1	1,33		
		Aga	1,20			Xrcc5	1,92	Pir	1,13			Ttr	1,33		
		Ergic1	1,18			Hgfac	1,92	Fgb	1,13			Bhmt	1,33		
		Itih4	1,18			Grn	1,91	Rbm3	1,13			Dnajc7	1,30		
		Ncl	1,17			Uqcc1	1,90	Ebp	1,12			LOC100912604	1,29		
		Cyp2c11	1,17			Serpinb1a	1,90	Cth	1,12			Acot13	1,29		
		Papss2	1,16			Lss	1,90	Fam114a1	1,12			Hsd17b8	1,27		
		Sqrdl	1,16			Commd7	1,90	Sult1e1	1,11			Acox1	1,27		
		C4a	1,13			Fscn1	1,89	Wars	1,11			Smpd1	1,27		
		Ass1	1,13			Kti12	1,89	Sars	1,11			Mgst3	1,27		
		Snrnp70	1,12			Gorasp1	1,89	Itih4	1,10			Col1a2	1,26		
		Aars	1,12			Pde2a	1,88	Lars	1,10			Serpina3k	1,26		
		Cyp1a2	1,11			Prmt1	1,87	Acss2	1,09			Wars	1,25		
		Ttc36	1,11			Sars2	1,85	Papss2	1,09			Alg3	1,24		
		Ebp	1,11			Trappc8	1,84	Iars	1,09			Fam98a	1,23		
		Ugdh	1,11			Fdps	1,83	Ttr	1,08			Ttll12	1,23		
		Hadhb	1,11			Tcf25	1,83	Ddc	1,08			Gpt	1,22		
		Acat1	1,10			Pnkd	1,82	Pign	1,08			Acadsb	1,21		
		Hadha	1,10			Pla2g15	1,82	Vps13c	1,08			Enpp3	1,21		
		Cyb5b	1,10			Nudt14	1,80	Banf1	1,08			Qsox1	1,21		
		Cth	1,09			Idi1	1,80	Eef1e1	1,07			Ube2g1	1,19		
		Sult1e1	1,09			Akap2	1,79	Dnph1	1,07			Sds	1,19		
		Eprs	1,09			Trmt1	1,78	Ybx1	1,07			Fndc3a	1,19		
		Gmppb	1,09			Spryd7	1,78	Tpr	1,07			Mpc1	1,18		
		Mgst3	1,09			Smarce1	1,77	Gpt	1,06			Mat2a	1,18		
		Slco1b2	1,08			Cyp2d4	1,77	Cyp1a2	1,06			Ilf3	1,18		
		Ldha	1,08			Tsr1	1,76	Hp	1,06			Sars	1,18		
		Cbs	1,08			Ankrd28	1,76	Dbi	1,06			A1i3	1,17		
		Ufl1	1,08			Aldh1a1	1,75	Fkbp11	1,06			Cstb	1,17		
		Npm1	1,04			Lztfl1	1,75	C9	1,05			Adprhl2	1,17		
		Ppp1ca	1,04			Rad21	1,75	Snap29	1,04			Iars	1,17		

		Gnmt	1,04			LOC100911860	1,74	Cyb5r3	1,04			Fam210a	1,16		
		Gpt	1,04			Cdc73	1,71	Pon3	1,04			Aldh4a1	1,16		
		Eif4a1	1,01			RGD1566134	1,71	Dhtkd1	1,04			Psap	1,16		
		Hnrnpa1	1,01			Zfpl1	1,70	Arl8b	1,03			Glul	1,16		
		Hpd	1,01			Gopc	1,70	Cad	1,03			Pycrl	1,16		
		LOC100365676	1,00			Ppip5k2	1,69	Mrps27	1,02			Ttc36	1,16		
						Nolc1	1,69	Lamtor3	1,02			Ivd	1,15		
						Ppt1	1,68	Reep5	1,02			Mat2b	1,14		
						Taok3	1,65	Pck1	1,02			Arpc5l	1,14		
						Abhd16a	1,65	Cyp2t1	1,01			Nubp1	1,13		
						Copz2	1,64	Enpp3	1,01			Abhd5	1,13		
						Mrps23	1,63	Fmo1	1,01			Nars	1,12		
						Crip1	1,62	Marcks	1,01			Sh3bgrl	1,12		
						Exoc5	1,62	Gys2	1,01			Lars	1,12		
						Tax1bp3	1,62	Gulo	1,01			Kmo	1,12		
						Mybbp1a	1,61	Chchd6	1,01			Gnl1	1,12		
						Inmt	1,60	Xpnpep2	1,01			Tmem14c	1,12		
						Dhcr24	1,59	Eif4g2	1,00			Apoa1	1,11		
						Wbp2	1,56	Rbm8a	1,00			Prkaa1	1,11		
						Mvd	1,55					Gmppa	1,11		
						Mvk	1,55					Srd5a1	1,11		
						Rdh2	1,54					Hadh	1,11		
						Cyp2c11	1,54					Pxmp2	1,11		
						Ppp3ca	1,53					Decr2	1,11		
						Hmgcs1	1,53					LOC100361144	1,10		
						Sds	1,52					Ass1	1,10		
						Sult1e1	1,52					Dus3l	1,10		
						Nme3	1,52					Pon3	1,09		
						Gstm3	1,49					Cad	1,09		
						Ttr	1,47					Gstm2	1,09		
						Acat2	1,47					LOC364561	1,09		
						Ttc36	1,47					Otc	1,09		
						Gpt2	1,45					Cyp2e1	1,09		
						Ldhd	1,45					Magt1	1,09		
						Ass1	1,44					Fads2	1,08		
						Grsf1	1,41					Eif3f	1,08		
						Cdk5	1,41					Ces1c	1,08		
						Sdhd.Lrrp1	1,41					Ndufa7l	1,08		
						Slc20a2	1,40					Papss2	1,07		
						Golga3	1,40					Coq9	1,07		
						Cars	1,38					Puf60	1,06		
						LOC100912604	1,37					Cat	1,06		
						Tecr	1,37					Atp5e	1,06		
						Ephx2	1,37					Eci1	1,06		
						Dhcr7	1,36					Atp5g3	1,05		
						Gtpbp3	1,35					LOC287167	1,05		
						Papss2	1,35					Fdx1	1,05		
						Dhtkd1	1,34					Ehhadh	1,04		
						Apoa2	1,33					Tecr	1,04		
						Smpd1	1,33					Anp32b	1,04		
						Tgm2	1,33					Fmo5	1,04		
						Serpina10	1,32					Hpd	1,04		
						Ces1d	1,32					Rpl10a	1,04		
						Apoc2	1,32					Xpnpep2	1,03		
						Alg3	1,32					Acadm	1,02		
						Ste	1,32					Cyb5a	1,02		
						Acss2	1,31					Pm20d1	1,02		
						Bhmt	1,31					Abcf2	1,02		
						Ndufa7l	1,30					Umps	1,02		
						Gstm2	1,29					Hint1	1,02		
						Mrpl27	1,28					Acsl1	1,01		
						Tpp1	1,28					Slc25a1	1,01		
						LOC299282	1,28					Sugct	1,01		
						Trabd	1,28					Tmem30a	1,01		
						Sars	1,28					Cyp2t1	1,01		
						Aldh4a1	1,28					Capza2	1,01		

						Gpt	1,26					H3f3c	1,00		
						Prodh	1,25					Glyat	1,00		
						Ptgs1	1,24					Psmb7	1,00		
						Slc25a1	1,23					Herc4	1,00		
						Hpd	1,23								
						Cbs	1,22								
						Acadsb	1,22								
						Ddc	1,22								
						Iars	1,22								
						Snap23	1,22								
						Asmtl	1,22								
						Gstt3	1,22								
						Anp32e	1,21								
						Dkc1	1,21								
						Hsd17b8	1,20								
						Pck2	1,20								
						LOC100361144	1,20								
						Rpl37a.ps1	1,19								
						Ctsa	1,19								
						Nap1l1	1,19								
						Coq9	1,19								
						Gstm1	1,18								
						Acot13	1,18								
						Nnmt	1,17								
						Gns	1,17								
						Fermt3	1,17								
						Bco2	1,17								
						Lars	1,17								
						Gulo	1,17								
						Pgpep1	1,16								
						Atxn10	1,16								
						Gsta4	1,16								
						Pcna	1,16								
						Ube2v2	1,15								
						Snrpd3	1,15								
						Cpt1a	1,15								
						Hsdl2	1,15								
						Cept1	1,14								
						Hsd17b13	1,14								
						Hsd11b1	1,13								
						Cmas	1,13								
						Magt1	1,13								
						Fetub	1,13								
						Fads2	1,13								
						Aldh1l1	1,13								
						Anp32b	1,12								
						Psap	1,12								
						Hexb	1,12								
						Fmo1	1,12								
						Mmaa	1,11								
						Nars	1,11								
						Fbl	1,11								
						Glul	1,11								
						Lamtor1	1,11								
						Pycrl	1,11								
						Gcat	1,10								
						Fkbp11	1,10								
						Cstb	1,10								
						Lclat1	1,10								
						Aass	1,10								
						Fam210a	1,10								
						Mpc1	1,10								
						Cbx1	1,09								
						Mgst3	1,09								
						Vamp8	1,09								
						Sco1	1,09								

						Mettl7b	1,08								
						Npc2	1,08								
						Gstk1	1,08								
						Ndufa6	1,08								
						Trim28	1,08								
						Umps	1,07								
						Eif3f	1,07								
						Kmo	1,07								
						Ivd	1,07								
						Nop58	1,07								
						Fam162a	1,07								
						Slc22a7	1,07								
						Adprhl2	1,06								
						Mat2b	1,06								
						Nucb1	1,06								
						Mt.cox3	1,06								
						Psmd9	1,06								
						Tmem30a	1,05								
						Cth	1,05								
						Rbx1	1,05								
						Pign	1,04								
						Glyat	1,04								
						Psmb7	1,04								
						Cyb5r3	1,04								
						Atp5e	1,04								
						Gnmt	1,04								
						Pon3	1,04								
						Otc	1,03								
						Marcks	1,03								
						Serpina3k	1,03								
						Gm2a	1,03								
						Tpmt	1,03								
						Hagh	1,03								
						Bcap31	1,02								
						Gnai2	1,02								
						Col1a2	1,02								
						Cyp2t1	1,02								
						Cyb5b	1,02								
						Gsta5	1,02								
						Rps27	1,01								
						Nat8	1,01								
						U2af2	1,01								
						Rpl38	1,01								
						Shpk	1,00								
						Suox	1,00								

Supplementary Table 4 *Metabolic pathways deregulated in the liver in experimental groups.*

groups	n	KEGG pathway	proteins	p-value
ENIL vs control	40	Terpenoid backbone biosynthesis	MVD, HMGCS2, HMGCS1, FDPS, MVK, IDI1, ACAT2, ACAT1	0.000
		Synthesis and degradation of ketone bodies	HMGCS2, HMGCS1, ACAT2, ACAT1	0.000
		Butanoate metabolism	HMGCS2, HMGCS1, ACAT2, ACAT1	0.001
		Valine, leucine and isoleucine degradation	HMGCS2, HMGCS1, ACAT2, ACAT1	0.006
PNIL vs control	134	Terpenoid backbone biosynthesis	MVD, HMGCS1, FDPS, MVK, IDI1, ACAT2, ACAT1	0.000
		Propanoate metabolism	LDHA, ACACB, ACSS2, ACAT2, ACAT1, HADHA	0.000
		Fatty acid metabolism	FADS1, PPT1, ACAT2, ACAT1, HADHA, HADHB	0.006
		Tryptophan metabolism	CYP1A2, ACAT2, ACAT1, HADHA, INMT	0.024
		Butanoate metabolism	HMGCS1, ACAT2, ACAT1, HADHA	0.038
		Valine, leucine and isoleucine degradation	HMGCS1, ACAT2, ACAT1, HADHA, HADHB	0.036
		Synthesis and degradation of ketone bodies	HMGCS1, ACAT2, ACAT1	0.047
		Steroid hormone biosynthesis	CYP2B2, CYP2D4, STE2, CYP2C12, CYP1A2, SULT1E1, CYP3A9, CYP2C11, HSD17B7	0.000
		Retinol metabolism	ALDH1A1, CYP2B2, CYP2C12, CYP1A2, CYP3A9, CYP2C11	0.032
		Chemical carcinogenesis	MGST3, CYP2B2, CYP2C12, CYP1A2, CYP3A9, CYP2C11	0.038
ENILOV vs control	33	no clusters found		
PNILOV vs control	305	Chemical carcinogenesis	GSTM1, MGST3, GSTM2, GSTM3, GSTA4, GSTA5, GSTK1, HSD11B1, CYP3A9, CYP2C11	0.004
		Glutathione metabolism	GSTM1, MGST3, GSTM2, GSTM3, GSTA4, GSTA5, GSTK1, LOC100912604	0.005
		Metabolism of xenobiotics by cytochrome P450	GSTM1, MGST3, GSTM2, GSTM3, GSTA4, GSTA5, GSTK1, HSD11B1	0.013
		Drug metabolism - cytochrome P450	GSTM1, MGST3, GSTM2, GSTM3, GSTA4, GSTA5, FMO1, GSTK1	0.013
PNIL vs ENIL	147	Pyruvate metabolism	ME1, ALDH1B1, PKLR, ACACB, PCK2, ACSS2, ACAT2, PCK1	0.000
		Glycolysis / Gluconeogenesis	GCK, ALDH1B1, HK3, PKLR, PCK2, ACSS2, PCK1	0.005
		Steroid hormone biosynthesis	CYP17A1, CYP2D4, STE2, CYP2C12, CYP1A2, SULT1E1, CYP3A9, HSD17B7	0.001
PNILOV vs ENILOV	198	Fatty acid metabolism	ACOX1, ACACB, ACADSB, ACSS2, ACSL1, ACADM, EHHADH, FADS1, FADS2, PPT1, HADH, TECR	0.000
		Fatty acid degradation	ECI1, ACOX1, ACADSB, ACSL1, ACADM, EHHADH, HADH	0.001
		PPAR signaling pathway	ACOX1, APOA2, ACSL1, APOA1, ACADM, EHHADH, FADS2, FABP5	0.002
		Drug metabolism - cytochrome P450	MGST3, FMO5, GSTM2, GSTM3, FMO1, CYP2E1	0.032
ENILOV vs ENIL	1	no clusters found		
PNILOV vs PNIL	97	no clusters found		

Gene names are listed according to HGNC guidelines (https://www.gennames.org).

Supplementary Table 5 Nutrition regime of experimental groups.

group	infusion	i.v. energy (kcal . day^{-1})	per os energy (kcal . day^{-1})	
			lipid emulsion	SD (granules)
CTRL	Plasmalyte	-	-	60
ENIL	Plasmalyte	-	20	40
ENILOV	Plasmalyte	-	20	40
PNIL	Intralipid	59.1	-	-
PNILOV	Intralipid + Omegaven (V:V/1:1.7)	59.2	-	-

SD, standard diet

Supplementary Table 6 Composition of parenteral nutrition mixture

Crude elements

Ingredients	kcal . kg-1 . day-1	% of non-amino acid energy
amino acid solution (Aminoplasmal 15%)	5	
glucose	140	70
lipid emulsion (Intralipid 20%)	60	30
or		
lipid emulsion (Intralipid 20% + Omegaven 10%)	60	30
total energy	205	

Amino acid composition

	mg . kg-1 . day-1
Isoleucine	47
Leucine	91
Lysine	64
Methionine	46
Phenylalanine	46
Threonine	43
Tryptophan	17
Valine	58
Arginine	128
Histidine	42
Glycine	154
Alanine	179
Proline	59
Aspartic acid	64
Cysteine	3
Glutamic acid	130
Serine	24
Tyrosine	4

Vitamins

	mg . kg-1 . day-1
Thiamin (B1)	124
Riboflavin B2)	140
Pyridoxine (B6)	155
Cyancobalamine (B12)	0.2
Nicotinamide	1552
Panthothenic acid	582
Ascorbic acid (C)	3880
Biotin	2.3
Folic acid	15.5

Electrolytes

	mg . kg-1 . day-1
Na+	15.6
K+	1.05
Mg2+	0.45
Ca2+	0.18
Cl-	16.1
SO42-	0.45
HPO42-	0.21
H2PO4-	0.11

Trace elements

	mg . kg-1 . day-1
Cr3+	0.008
Cu2+	0.233
Fe3+	0.776
Mn2+	0.039
Mo6+	0.008
Se4+	0.039
Zn2+	2.988
I-	0.039
F-	1.940

Supplementary Table 7 *Primer sequences.*

Gene	Forward Primer	Reverse Primer	ID gene
Hmox1	ACATTGAGCTGTTTGAGGAGCTG	GTCGCCAACAGGAAACTGAG	NM_012580.2
Nqo1	GGTGATATTTCAGTTCCCATTG	AGACCTGGAAGCCACAGAAG	NM_017000.3
Gclc	GAGAGCATCTGGAGAACTAATGAC	TCTTCACAGCGTCTGAGTGG	NM_012815.2
Ccr-2	TTTGATCCTGCCCCTACTTG	AACGCAGCAGTG AACGCAGCAGTGTGTCATTC	NM_021866.1
Il8	CGAAGTCATAGCCACACTC	AGGACCCTCAATAGAAATCG	NM_030845.1
Il1b	AGTCTGCACAGTTCCCCAAC	GAGACCTGACTTTGGCAGAGG	NM_031512.2
Tnfa	CAAGGAGGAGAAGTTCCCAAATG	GCTTGGTGGTTTGCTACGAC	NM_012675.3
B2M	CCATTCAGAAAACTCCCCAAATTC	GGCTCCTTCAGAGTGACGTG	NM_012512.2
Cyc 1	GGGACTCAAGATGTTGTTGATG	GTTCCTGGTCTGCTGAGAGC	NM_001277194.1
Alox5	TTGGCCAGTATGACTGGTGCTC	CTCCAGGTTCTTGCGGAATC	NM_01288.1
Ptges	GGAAGAAGGCTTTTGCCAAC	TGGGTCCCAGGAATGAGTAG	NM_021583.3
Ptgis	CTGTGCTAGACAGTGTGCTC	GATTTCTGGGTCCTTCTGG	NM_031557.2
Ptgs2	TGTTGACGTCCAGATCAC	TCCTCAGAAGAACCTTTTCC	NM_017232.3
Il4	GGCAACAAGGAACACCAC	AGGACATGGAAGTGCAGGAC	NM_201270.1
IL6	CTCTCCGCAAGAGACTTCCAG	ACAGTGCATCATCGCTGTTC	NM_012589.1
